# Spontaneous Orientation Polarization of Anisotropic Equivalent Dipoles Harnessed by Entropy Engineering for Ultra-Thin Electromagnetic Wave Absorber

**DOI:** 10.1007/s40820-024-01507-0

**Published:** 2024-09-26

**Authors:** Honghan Wang, Xinyu Xiao, Shangru Zhai, Chuang Xue, Guangping Zheng, Deqing Zhang, Renchao Che, Junye Cheng

**Affiliations:** 1 Liaoning Key Lab of Lignocellulose Chemistry and BioMaterials, Liaoning Collaborative Innovation Center for Lignocellulosic Biorefinery, School of Light Industry and Chemical Engineering, Dalian Polytechnic University, Dalian, 116034 People’s Republic of China; 2 School of Life Science and Biotechnology, Dalian University of Technology, Dalian, 116024 People’s Republic of China; 3 Department of Mechanical Engineering, Hong Kong Polytechnic University, Hung Hom, Kowloon, Hong Kong, 999077 People’s Republic of China; 4 School of Materials Science and Engineering, Qiqihar University, Qiqihar, 161006 People’s Republic of China; 5 Laboratory of Advanced Materials, Shanghai Key Lab of Molecular Catalysis and Innovative Materials, Academy for Engineering & Technology, Fudan University, Shanghai, 200438 People’s Republic of China; 6 Department of Materials Science, Shenzhen MSU-BIT University, Shenzhen, 517182 People’s Republic of China

**Keywords:** High-entropy alloys, Carbothermal shock, Switchable electron migration modes, Emblematic shell-core heterointerfaces, Ultra-thin thickness

## Abstract

The synthesis of carbon supporter/nanoscale high-entropy alloys (HEAs) electromagnetic response composites by carbothermal shock method has been identified as an advanced strategy for the collaborative competition engineering of conductive/dielectric genes. Electron migration modes within HEAs as manipulated by the electronegativity, valence electron configurations and molar proportions of constituent elements determine the steady state and efficiency of equivalent dipoles. Herein, enlightened by skin-like effect, a reformative carbothermal shock method using carbonized cellulose paper (CCP) as carbon supporter is used to preserve the oxygen-containing functional groups (O·) of carbonized cellulose fibers (CCF). Nucleation of HEAs and construction of emblematic shell-core CCF/HEAs heterointerfaces are inextricably linked to carbon metabolism induced by O·. Meanwhile, the electron migration mode of switchable electron-rich sites promotes the orientation polarization of anisotropic equivalent dipoles. By virtue of the reinforcement strategy, CCP/HEAs composite prepared by 35% molar ratio of Mn element (CCP/HEAs-Mn_2.15_) achieves efficient electromagnetic wave (EMW) absorption of − 51.35 dB at an ultra-thin thickness of 1.03 mm. The mechanisms of the resulting dielectric properties of HEAs-based EMW absorbing materials are elucidated by combining theoretical calculations with experimental characterizations, which provide theoretical bases and feasible strategies for the simulation and practical application of electromagnetic functional devices (e.g., ultra-wideband bandpass filter).

**Supplementary Information:**

The online version contains supplementary material available at 10.1007/s40820-024-01507-0.

## Highlights

- The strengthening mechanism of spontaneous orientation polarization of anisotropic equivalent dipoles within high-entropy alloys (HEAs) is proposed for enhancing dielectric attenuation of HEAs.
- The source of carbon supporter is expanded to the biomass category, which can construct the shell-core heterointerfaces with HEAs by means of a reformative carbothermal shock method.
- The sample carbonized cellulose paper/HEAs-Mn_2.15_ achieves efficient electromagnetic wave absorption of -51.35 dB at an ultra-thin thickness of 1.03 mm.
- This work combines theoretical calculations and electromagnetic simulations to propose feasible strategies for the design and application of electromagnetic functional devices such as ultra-wideband bandpass filter.

**Supplementary Information:**

The online version contains supplementary material available at 10.1007/s40820-024-01507-0.

## Graphical abstract

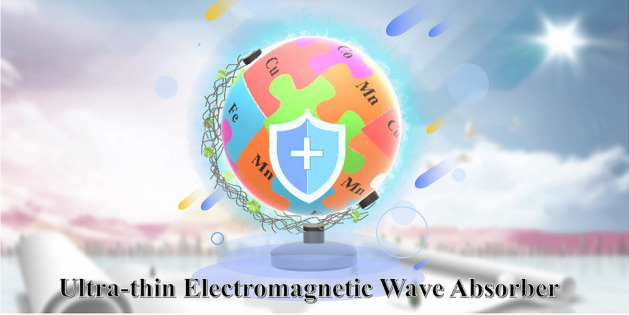

## Introduction

The reprogramming of multiple constituent elements of materials is expected to trigger a qualitative gene change in the lattice structure, which could be caused by chemical disorder or high-entropy (HE) effect [1–3]. Typically, the Gibbs free energy of materials is minimized by the accumulation of high configurational entropy through the ingenious fusion of five or more elements (with molar ratios ranging from 5%–35%), resulting in a single-phase solid solution at equilibrium where multi-components exhibit an unexpected combination of physical and chemical properties [4–6]. As of today, the concept of HE is feasibly integrated in various scientific research topics, such as HE alloys (HEAs), HE metal oxides (HEOs), HE sulfides (HESs), HE MXene (HEM) and HE ceramics (HEC) that have been widely developed in the field of tribology [7], energy conversion and storage [8], solar energy catalysis [9], biomedicine [10], irradiation response [11]. It is of much interest that the concept of HE is applied to develop materials with high performance for electromagnetic (EM) wave attenuation. In general, optimization of EM parameters of evolution electromagnetic wave (EMW) absorbing materials is achieved by a flexible synergy of soft magnetic properties [12], multiphase interfaces, lattice distortion effects, unsaturated coordination and other defect engineering [13] to relieve EMW pollution [14–16] and develop EMW shielding devices [17, 18]. Recently, based on an electron migration mechanism as proposed by our group, FeCoNiCuZn HEAs have been used to achieve low-frequency EMW absorption [19]. Equivalent dipoles induced by double electron-rich sites (with Cu and Zn elements) act as effective attenuation units, resulting in an increased threshold of the maximum critical state for dynamic equilibrium between impedance matching and attenuation constants in the low-frequency region. This mechanism is different from the HE effects of HEAs and provides innovative ideas for improving dielectric properties of HEAs-based EMW absorbing materials.

In order to further enhance the equivalent dipole-induced polarization effect within HEAs, it is necessary to tune the electron migration mode, which can be achieved through reprogramming the constituent elements and their molar ratios. HEAs with face-centered cubic (FCC) crystal structure that are composed of elements Fe, Co, and Ni possess excellent physical properties such as good tensile ductility, fracture toughness and shock resistance, and are promising candidates for EMW absorption [20–22]. The selection of other coordination elements is mainly based on screening of atomic radius, electronegativity, valence electron configuration, and *d*-band center [23–25]. The selection of constituent elements with similar atomic radius and differentiated electronegativity randomly arranged in the FCC lattice is a necessary prerequisite for the generation of equivalent dipoles. Metal elements with *d*-band centers far from the Fermi level usually act as electron-rich sites, whose fettering electron ability determines the equivalent dipole efficiency. The mismatched atomic radius and stable valence electron of Zn lead to a decreased work function of FeCoNiCuZn HEAs, and electron losing tendency bury the foreshadowing for the annihilation of equivalent dipoles [26, 27]. Taking into account its strong fettering electron ability of Cu, Cu is considered as a single electron-rich site, and Mn with a similar atomic radius and a differentiated electronegativity is the preferred alternative element to stimulate the uneven distribution of charge. Furthermore, Mn with a high molar ratio will be more closely distributed around Cu, not only promoting the generation of oriented equivalent dipoles, but also catalyzing nucleation of mixed liquid metals through a process of “fission–fusion”. For the sake of high configurational entropy, the maximum molar ratio of element Mn should not exceed 35%.

The montage of xenogeneic dielectric genes is an antecedent strategy for optimizing EMW absorption parameters [28–30]. A carbothermal shock method dominated by carbon “metabolism” can be used to construct a nanoscale HEAs/carbon supporter coupling system with an electron/dipole collaborative competition mechanism [31, 32]. Carbothermal shock method has unique advantages for the preparation of HEAs: Firstly, it is an ultra-convenient preparation process that enables the nucleation of HEAs within millisecond shock durations. Secondly, the ultra-rapid heating and cooling rates contribute to the formation of nanoscale HEAs. Finally, the carbon support and ultra-high temperature can inhibit the oxidation of metals with high oxidation potentials. The residual oxygen-containing functional groups (O·) of carbon supporters play an irreplaceable role in regulating electron transport and dipole polarization, as well as acting as nucleation sites of HEAs. However, the carbon supporters where transient high-voltage currents are passed through have little opportunity in retaining abundant O·[15]. Inspired by the skin effect, an external superconducting accessory, which can provide a protective layer for preventing the carbon supporters from direct contact with currents, is used in this work. The upgrading carbothermal shock method provides abundant access to carbon sources [33], including those of biomass carbon with low conductivity and multifunctional groups, which fulfills the strategic of “carbon peaking and carbon neutrality” [34, 35]. In this work, carbonized cellulose paper (CCP)/FeCoNiCuMn HEAs (with 35% molar ratio of Mn) hybrids (CCP/HEAs-Mn_2.15_) with emblematic shell-core heterointerfaces are prepared via the reformative carbothermal shock method and through using Mn, which has strong catalytic activity. The outstanding EMW absorption performance at an ultra-thin thickness of 1.03 mm is attributed to the electron migration mode with single electron-rich site, which possesses intense fettering electron ability to generate localized equivalent dipoles. The facile carbothermal shock method and rich electron migration modes provide design freedom under the reliable dielectric-response mechanisms for the development of HEAs-based functional devices with excellent EMW absorption performance.

## Experiment Section

### Materials

The anhydrous FeCl_3_, CoCl_2_, NiCl_2_, CuCl_2_, ZnCl_2_, MnCl_2_ and ethanol were purchased from Shanghai Aladdin Biochemical Technology Co. Ltd. All reagents are analytically pure.

### Fabrication of CCP/MMSs, CCP/HEAs-Zn, CCP/HEAs-Mn, CCP/HEAs-Mn_2.15_

The mixture of metal salts with desired molar ratios was dissolved in ethanol under ultrasonic treatment to prepare three precursor metal salt solutions (0.5 mol L^−1^) denoted as Solution A, Solution B, and Solution C. The Solutions A for the synthesis of CCP/MMSs and CCP/HEAs-Zn contain equal molar ratios (0.1 mol L^−1^) of FeCl_3_, CoCl_2_, NiCl_2_, CuCl_2_, ZnCl_2_. The Solutions B for the synthesis of CCP/HEAs-Mn contain equal molar ratios (0.1 mol L^−1^) of FeCl_3_, CoCl_2_, NiCl_2_, CuCl_2_, MnCl_2_, while the Solutions C for the synthesis of CCP/HEAs-Mn_2.15_ contain non-equal molar ratios of FeCl_3_ (0.08125 mol L^−1^), CoCl_2_ (0.08125 mol L^−1^), NiCl_2_ (0.08125 mol L^−1^), CuCl_2_ (0.08125 mol L^−1^), MnCl_2_ (0.175 mol L^−1^). 0.5 mL of Solution A were dropped onto two CCP (the specific preparation process is shown in S1.1 of the supporting information) with the same size and dried at room temperature in a N_2_-filled glove box, while the same operations were carried out with Solution B and Solution C. Four completely dry samples were subjected to transient ultra-high temperature treatment in a reformative carbothermal shock equipment with a high conductivity tube. The shock temperature was uniformly set to be 1873 K. When the shock duration was 50 ms, the obtained sample was labeled as CCP/MMSs. When the shock duration was 100 ms, the obtained samples were labeled as CCP/HEAs-Zn, CCP/HEAs-Mn, CCP/HEAs-Mn_2.15_, respectively. The relevant information about Mn, Fe, Co, Ni, Cu, Zn is shown in Table S1.

## Results and Discussion

### Construction of CCP/HEAs System

**Fig. 1:**
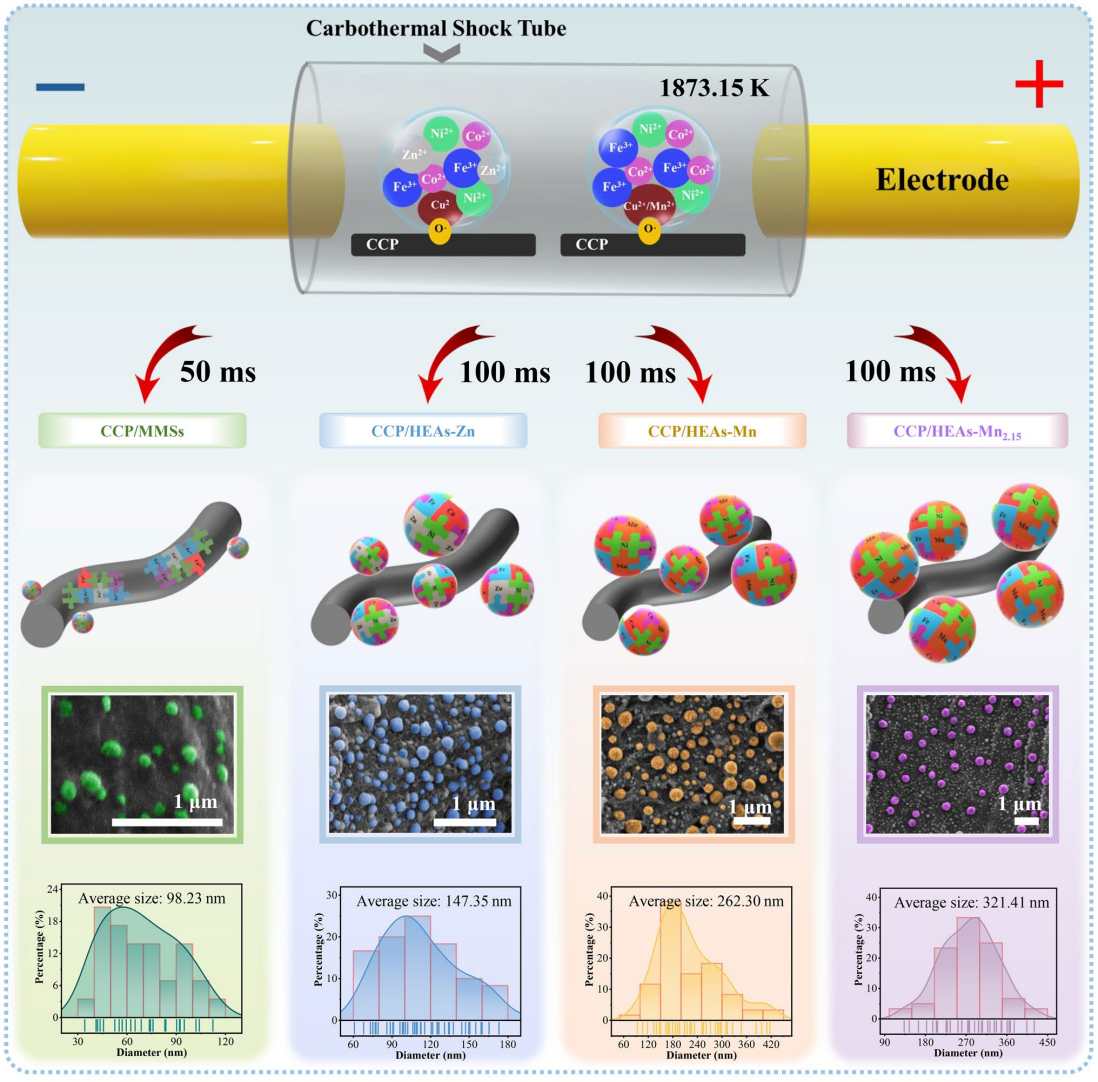
Schematic of reformative carbothermal shock process for the synthesis of CCP/MMS, CCPHEAs-Zn, CCP/HEAs-Mn and CCP/HEAs-Mn_2.15_, and corresponding SEM images and particle size distribution statistics

In view of the fact that most of metal elements are immiscible under thermodynamic equilibrium conditions, there are some pioneer investigations that the carbothermal shock method has been used to synthesize HEAs nanoparticles containing five or more metal elements [36, 37]. Conventional carriers for carbothermal shock method are usually highly conductive carbon materials that provide extremely high temperature (> 1773 K) with millisecond shock durations, where the metal elements are condensed into solid solutions at an ultra-fast cooling rate (> 800 K s^−1^). However, excessive conductivity is not conducive to achieving a dynamic balance between impedance matching and attenuation constants in EMW absorbing materials. Herein, a reformative carbothermal shock method is proposed, whose supporter is selected to be biomass carbon CCP and is equipped with a conductive carbothermal shock tube. When a voltage is applied, the current will be conducted along the low resistance path of the as-built carbothermal shock tube, providing thermal energy required for CCP carbonization, while the annealing process without the direct contact to current retains the abundant O· in the carbonized cellulose fiber (CCF). The reformative process not only generates dipoles that can promote relaxation loss for EMW attenuation, but also provides sufficient active sites for the synthesis of HEAs nanoparticles. The typical samples are CCP/FeCoNiCuZn (with equimolar ratio) mixed metal salts (CCP/MMSs), CCP/FeCoNiCuZn HEAs (with equimolar ratio) hybrids (CCP/HEAs-Zn), CCP/FeCoNiCuMn HEAs (with equimolar ratio) hybrids (CCP/HEAs-Mn), and CCP/HEAs-Mn_2.15_. The configurational entropy of HEAs-Zn, HEAs-Mn and HEAs-Mn_2.15_ are shown in Table S2. As shown in Fig. 1, with the intervention of high molar ratio of Mn, the size of HEAs nanoparticles gradually increases and tends to be homogenized and uniformly distributed on the CCF, which could be related to the catalytic activity of metal element. More importantly, the crystal structure and electron migration of HEAs as well as the microstructure and defect concentration of CCF are systematically investigated [38], based on which a theoretical framework for exploring the mechanisms of nucleation of HEAs and EMW attenuation has been established.

### Morphology, Structure, and Chemical State Characterization

**Fig. 2:**
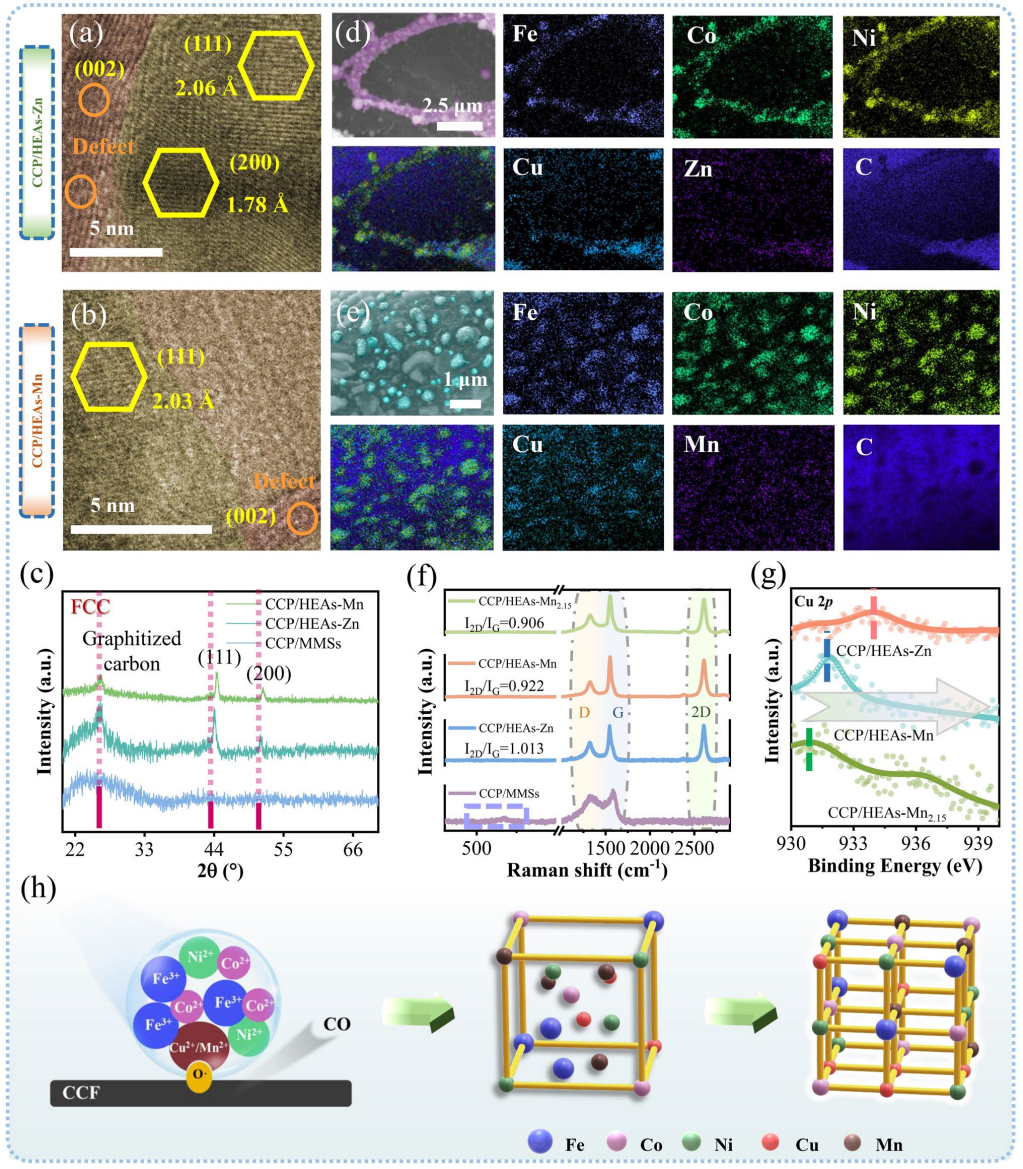
The HRTEM images of **a** CCP/HEAs-Zn and** b** CCP/HEAs-Mn; **c** XRD patterns of CCP/MMSs, CCP/HEAs-Zn and CCP/HEAs-Mn; Elemental mapping images of **d** CCP/HEAs-Zn and** e** CCP/HEAs-Mn; **f** Raman spectra of CCP/MMSs, CCP/HEAs-Zn, CCP/HEAs-Mn and CCP/HEAs-Mn_2.15_; **g** The binding energy of Cu 2*p* in CCP/HEAs-Zn, CCP/HEAs-Mn and CCP/HEAs-Mn_2.15_; **h** The nucleation mechanism of HEAs nanoparticles

As one might expect, neighboring transition metal elements with similar thermodynamic properties located in the same period are prone to forming entropy-stabilized solid solution [39, 40]. Through the cooperation of Zn and Mn with basic elements Fe, Co, Ni, and Cu, the conventional thermodynamic equilibrium is disrupted under the powerful driving source of thermal energy, resulting in a highly disordered atomic arrangement state. The differences in the types and molar ratios of the constituent elements enable a wide range of highly designable gene sequences. During a reformative carbothermal shock process, designative metal elements with different atomic radius, reduction potentials, and crystal structures are readily “condensed” into nuclei with the classical FCC phase of metals (Fig. 2a–c). The (111) and (200) crystal planes of CCP/HEAs-Zn and CCP/HEAs-Mn attributed to the FCC crystal structure of HEAs can be clearly identified in HRTEM images and XRD patterns. Since the atomic radius of Mn is similar to Fe, Co, Ni, and Cu, which is smaller than Zn, the atomic radius difference of HEAs-Mn (0.69%) is smaller than that of HEAs-Zn (3.03%), which is confirmed by Formula (14) in Supporting Information. The suppressed lattice distortion leads to a smaller lattice constant for HEAs-Mn, thus the XRD characteristic peaks of CCP/HEAs-Mn is shifted to the high angles compared to CCP/HEAs-Zn. The relatively strong reduction activity of Mn not only intensifies the fission/fusion behavior of metals but also increases the crystallinity of CCP/HEAs-Mn. The structural configuration of CCP/HEAs-Zn and CCP/HEAs-Mn at high temperatures is more inclined to the formation of FCC structure under low strains, which is attributed to the decrease of Gibbs free energy as driven by the entropy increment. Furthermore, there is an obvious peak between 200 and 1000 cm^−1^ in the Raman spectra of CCP/MMSs rather than CCP/HEAs-Zn, CCP/HEAs-Mn and CCP/HEAs-Mn_2.15_ (Fig. 2f), proving that almost no corresponding metal oxides are produced in the systems with high entropy, ensuring that the dynamic chemical equilibrium of crystal structure and valence states of constituent elements in the systems are maintained [19].

The shock temperature (1873.15 K) is set to be higher than the melting point of metal precursor corresponding to each metal salt, enabling that the metal elements with a liquid phase flow freely within an extremely short shock time (100 ms), accompanied by the release of gases. This process will exacerbate the coarsening of metals to reduce the surface energy, while the intervention of Cu and Mn with catalytic activity will promote the coalescence and nucleation of different metals. As a result, the formation of nanoscale HEAs is not likely to occur. Interestingly, SEM–EDS element mappings show that the surface of CCF is densely occupied by nanoscale HEAs-Zn and HEAs-Mn, and those elements in the nanoparticles are uniformly distributed without significant elemental segregation (Fig. 2d, e). In order to elucidate the formation mechanism of HEAs nanoparticles, a case-by-case ranking of defect concentration of carbon carriers is carried out (Fig. 2f). Obviously, the graphitization degree of CCF in each sample varies within a reasonable range under the same shock temperature. The transient shock time (50 ms) of the inchoate technological process results in CCF of CCP/MMSs, which is characterized to be amorphous carbon with small graphite domains, as demonstrated by the existence of unsharp and widened peak attributed to the (002) crystal plane in the XRD patterns and the absence of 2D peak in Raman spectra. Consequently, the increased entropy of mixing does not lead to the formation of HEA nanoparticles, with evidences of undetected characteristic XRD peaks of HEAs (Fig. 2c) and uneven elemental distribution (Fig. S1). By virtue of the prolonged shock duration (100 ms) as controlled by electrical pulse parameters, the graphitization degrees of CCF are significantly enhanced, among which the value of *I*_2D_/*I*_G_ for Raman characteristic peaks of CCP/HEAs-Zn is the highest, while that of CCP/HEAs-Mn_2.15_ is the lowest, which is accompanied by the widened full width at half maximum of the Raman peaks, suggesting that the CCF surface of CCP/HEAs-Mn_2.15_ carrying more O·has abundant defects. Figure 1b shows that the HEAs nanoparticles in CCP/HEAs-Mn_2.15_ with more regular spherical shape are uniformly distributed and tend to have homogeneous sizes, which are presumed to be positively correlated with the defect concentration of CCF and reduction activity of metals.

The nucleation mechanisms of HEAs nanoparticles can be revealed by redox reactions involving the carbon supporter CCF, O· and metals with strong reduction activity (Fig. 2h). Liquid-phase metals at high temperatures autonomously aggregate at O· active sites to reduce the activation energy of redox reactions, and high concentrations of O· increase the fluidity of mixed liquid, which can be driven to collide and fuse violently, alleviating HEAs nanoparticles coarsening. Oxygen-containing groups that are not fully reduced are considered as dipoles with EM response, which are important factors for dielectric genes. Moreover, the carbon supporter CCP used in carbothermal shock method can be considered as a 3D network built by CCF whose diameters are constrained to be ~ 500 nm, while each relatively independent CCF can be considered as a microscopic 1D free electron transport channel to induce conductive loss. It follows that carbon supporter with defects is an important medium for synthesizing HEAs nanoparticles and assisting in EM attenuation. It is worth noting that the graphitization degree of CCF is affected by the current and its directionality. The reformative equipment accesses omni-directional conductive pathways for resistive CCP, which provides prodigious amounts of heat to CCP by inducing current conducting along surface of conductive pathways while suppressing the transient graphitization of CCF, and facilitates the retention of defects of CCF located in the non-conductive pathways. In addition, differences in the graphitization degree of CCF are related to the electron migration modes within HEAs. As shown in Fig. 2g, the binding energy of Cu 2*p* in CCP/HEAs-Mn_2.15_ shifts towards low energy level, indicating that the electron migration effect in HEAs-Mn_2.15_ is enhanced and stable dipoles are formed. Meanwhile, the migration of current towards the CCP is inhibited by the enhanced binding-electron ability of HEAs-Mn_2.15_, protecting the O· of CCP to induce more HEAs to nucleate and enhance dipole polarization. Therefore, the electron migration mechanism within HEAs is the theoretical basis for revealing the electron-rich polarization effect and the dielectric properties of CCF. The enhanced electron migration effect within HEAs is a preferred strategy to increase the defect concentration of CCP to promote HEAs nucleation and dipole polarization.

### Equivalent Dipole Efficiency Within HEAs with Different Components

**Fig. 3:**
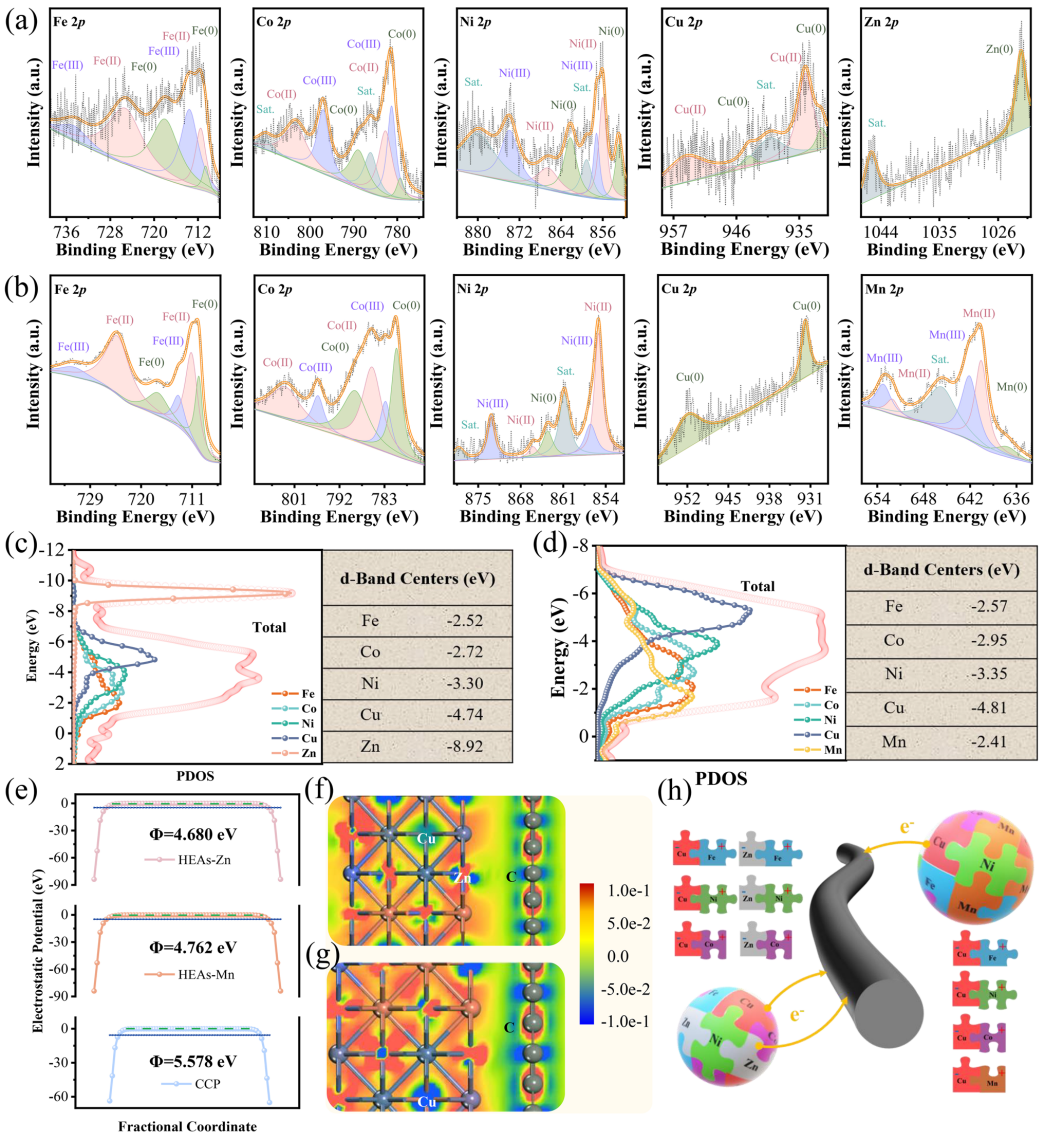
XPS spectra of **a** CCP/HEAs-Zn and **b** CCP/HEAs-Mn; The *d*-orbital PDOS of Fe, Co, Ni, Cu, Zn, Mn and the total value for **c** CCP/HEAs-Zn and **d** CCP/HEAs-Mn; **e** Work function of CCP/HEAs-Zn, CCP/HEAs-Mn and CCP; The differential charge density of **f** CCP/HEAs-Zn and **g** CCP/HEAs-Mn heterointerfaces; **h** Equivalent dipoles within HEAs

The transient nucleation process at ultrahigh temperature leads to the integration of anisotropic metallic elements from a chaotic state into a stable state, involving the adjustment of the electronic structure and charge distribution of each metallic element, which can be appropriately reflected by XPS spectroscopy. The specific 0-valence characteristic peaks attributed to metal elements of HEAs are shown in Fig. 3a, b, confirming that each metal element fused in the HEAs tends to regress to Metal(0) state by gaining or losing electrons, which is not observed in CCP/MMSs where HEAs have not been formed (Fig. S2). The binding energies of Fe 2*p*, Co 2*p* and Ni 2*p* in CCP/HEAs-Zn are higher than that of corresponding Metal(0) states and shift to higher energy levels, while Cu 2*p* and Zn 2*p* have the opposite trend, proving that Cu and Zn can be considered as double electron-rich sites (Fig. S3a). The difference is that only the binding energy of Cu 2*p* in CCP/HEAs-Mn is lower than that of corresponding Metal(0) state (Fig. S3b). Electron migration modes with single electron-rich sites have a conceivable impact on HEAs-Mn, in terms of localized electron density and electron escape capability. The differential charge density is a visualization form that can cast light on the localized electron redistribution around different metal elements, verifying electron migration modes with single/double electron-rich sites. The high-charge regions in blue representing electron-accumulation attributed to Cu and Zn and the low-charge regions in red representing electron-divergence attributed to Fe, Co, Ni, Mn (Fig. 3f, g). The systematic research of valence electron arrangement of each metal element is necessary, in order to provide a rational explanation for the switching of electron migration modes.

The valence electrons of transition metals occupy the *s*, *p*, and *d* orbitals, and the energy band broadening of *d* orbital is a decisive factor that determines the electronic properties of transition metals. The metal catalysts are adsorbed to the O· active sites on CCF surface to promote redox reactions, which involve rearrangement of valence electron orbitals and preferential electron filling, and can be interpreted by *d*-band center theory (Fig. 3c, d). The half-filled *d*-orbital of Mn has the highest *d*-band center position, resulting in its antibonding orbitals crossing the Fermi level that are not prone to electron filling, which facilitates the *sp*-*d* orbital hybridization between $\text{O}^{.}$ and Mn. Briefly, the incorporation of Mn accelerates the high-entropy alloying process. While Cu and Zn with fully-filled *d*-orbitals have the lowest *d*-band center positions, which promote the completion of high-entropy alloying by collecting free electrons. The *d*-band centers of Fe, Co, Ni, and Cu in CCP/HEAs-Zn are shifted downwards compared to those in CCP/HEAs-Mn, which are attributed to the weakening of interatomic interaction due to the increased lattice spacing of CCP/HEAs-Zn. The switching of the electron migration modes verified by the deviation of *d*-band center positions also induces the differentiation of work functions between CCP/HEAs-Zn and CCP/HEAs-Mn. Based on the Hund rule, electrons flowing towards the Cu sites will be riveted, while those flowing towards the Zn sites will run off, resulting in the work function of CCP/HEAs-Mn larger than that of CCP/HEAs-Zn. Both of the work functions are lower than that of conductive CCF (Fig. 3e). The flowing electrons confined within the HEAs will migrate directionally in an alternating EM field to generate localized equivalent dipoles (Fig. 3h). The mixed metal salts with stable valence states in CCP/MMSs lack localized electron migration for the formation of equivalent dipoles. Consequently, the dielectric loss of the resulting material is only attributed to functional group polarization and conductive loss of CCF.

### Optimized Strategy for Ultra-Thin EMW Absorption

**Fig. 4:**
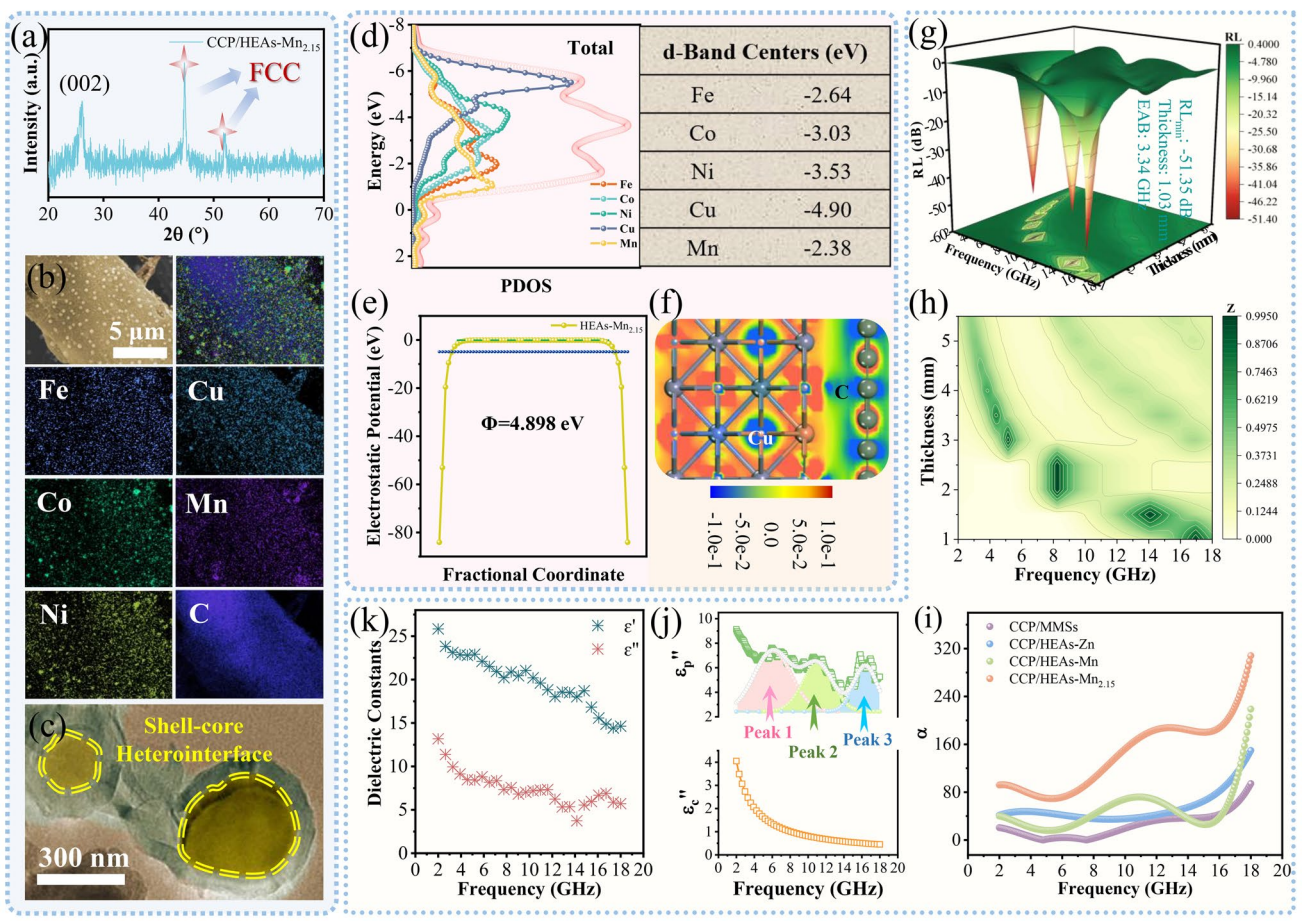
**a** XRD patterns, **b** elemental mapping images, **c** TEM images, **d** *d*-orbital PDOS of Fe, Co, Ni, Cu, Mn and the total value, **e** work function of CCP/HEAs-Mn_2.15_; **f** The differential charge density of CCP/HEAs-Mn_2.15_ heterointerfaces; **g** 3D RL plot, **h** M_Z_, **i** *α*, **j** *ε*_*p*_″ and *ε*_*c*_″ and **k** ε′ and ε″ of CCP/HEAs-Mn_2.15_

Taking into account the determinants of localized dipolar polarization, including the electron migration modes with single electron-rich sites dominated by Cu, the lowest binding energy of Cu 2*p* (Figs. 2g, S4 and S5) and the highest defect concentration of CCF (Fig. 2f) in CCP/HEAs-Mn_2.15_, HEAs-Mn_2.15_ is presumed to have the most promising dipolar polarization module. In addition to the classical FCC crystal structure (Fig. 4a) and non-segregated element distribution (Fig. 4b) attributed to HEAs-Mn_2.15_, the emblematic shell-core heterointerfaces are shown in the TEM image of CCP/HEAs-Mn_2.15_ (Fig. 4c). The formation of the customized encapsulating-type CCF is attributed to the metabolic process of amorphous carbon. The abundant O· active sites of CCF are reduced by the metal catalysis and the CO gas could be release, which drives the CCF to curl along the outer wall of HEAs-Mn_2.15_. The differential conductivity inside and outside the shell-core heterointerfaces promotes interfacial polarization. The electron migration mode with single electron-rich sites is verified by the *d*-band center position of each metal element (Fig. 4d) and the differential charge density of CCP/HEAs-Mn_2.15_ (Fig. 4f). Compared with CCP/HEAs-Mn, when the molar ratio of Mn is increased, the *d*-band of Mn in CCP/HEAs-Mn_2.15_ is broadened, resulting in a downward shift of *d*-band center, which implies that Mn is more readily adsorbed by O· to promote the homogenization of HEAs-Mn_2.15_ nanoparticles. While an upward shift of the *d*-band centers of Fe, Co, Ni, and Cu intensifies the demand for free electrons to accomplish the conversion to Metal(0) states, which is reflected by the increased work function of CCP/HEAs-Mn_2.15_ (Fig. 4e). Furthermore, the increase in molar ratio of Mn enhances the probability that Mn encountering the Cu electron-rich sites, and their electronegativity difference induces the formation of more persistent and stable equivalent dipoles.

For materials without magnetic loss (Fig. S6), competitive collaboration between conductive gene and polarization gene reveals the structure–activity relationship between microstructure engineering and EM response devices [41, 42]. The multidimensional comprehensive evaluation of EMW absorption performance bores down on the absorption intensity, matching thickness and effective absorption bandwidth (EAB). Critical conditions for dynamic equilibrium between impedance matching and attenuation constants are determined by localized electron migration modes. The EMW absorption performances of CCP/MMSs (*RL*_min_ is − 42.2 dB with a thickness of 4.01 mm and an EAB of 1.0 GHz, as shown in Fig. S7), CCP/HEAs-Zn (*RL*_min_ is − 59.5 dB with a thickness of 4.00 mm and an EAB of 2.5 GHz, as shown in Fig. S8), CCP/HEAs-Mn (*RL*_min_ is -76.2 dB with a thickness of 5.04 mm and an EAB of 1.2 GHz, as shown in Fig. S9), CCP/HEAs-Mn_2.15_ (*RL*_min_ is − 51.4 dB with a thickness of 1.03 mm and an EAB of 3.4 GHz, as shown in Fig. 4g) are gradually optimized. The localized dipolar polarization units with the strongest attenuation ability in CCP/HEAs-Mn_2.15_ (Fig. 4i) enables its impedance to match with that of free space at an ultra-thin thickness (Fig. 4h). The introduction and modulation of simple components carbon support and HEAs strengthen the customized EM response modes, which is conducive to the optimization of the absorption intensity and matching thickness at specific frequencies. Although CCP/HEAs-Mn_2.15_ does not possess broadband electromagnetic wave absorption properties, the optimization strategy of the dielectric properties of HEAs and the ultra-convenient and green preparation method are elaborated in depth through the well-designed enhancement mechanism of the equivalent dipole polarization effect. In addition, all samples equipped with CCF carbon supporters show three-band EMW absorption characteristics, whose matching thickness are decreased from 9.0 to 5.5–6.0 mm by the efficient synthesis of HEAs, which is attributed to the response of multiple loss mechanisms of EMW in different bands. The relevant dielectric properties are usually defined by the dielectric constants, where the real part (ε′) represents the storage capacity of electric energy and the imaginary part (ε″) represents the dissipation capacity of electric energy, both exhibiting a dependence on frequency (Fig. 4k). The largest tanδε (ε″/ε′) value of CCF/HEAs-Mn_2.15_ exhibits the strongest dielectric loss degree (Fig. S13). According to the classical Debye theory, the dissipative capacity is attributed to a synergistic effect of ε_c_″ and ε_p_″, which represent the conductive genes and polarization genes, respectively. The ε_c_″ curve is related to the conductivity of the sample (Fig. S14). The ε_p_″ curve is fitted by three characteristic relaxation peaks attributed to the resonance behaviors of residual O· in CCF, the shell-core heterointerfaces of CCF/HEAs-Mn_2.15_ and the equivalent dipoles induced by localized electron migration inside HEAs-Mn_2.15_, respectively (Fig. 4j). Meanwhile, more Cole–Cole semicircles and longer polarization relaxation time attributed to multi-polarization relaxation process of CCF/HEAs-Mn_2.15_ are shown in Figs. S10-S13. It is evident that the switching and intensification of localized electron migration modes have enabled controllable preparation of ultra-thin EMW absorption materials based on the optimized EM parameters and enhanced EM attenuation capability induced by the coupled system of biomass-derived carbon CCF and HEAs-Mn_2.15_.

**Fig. 5:**
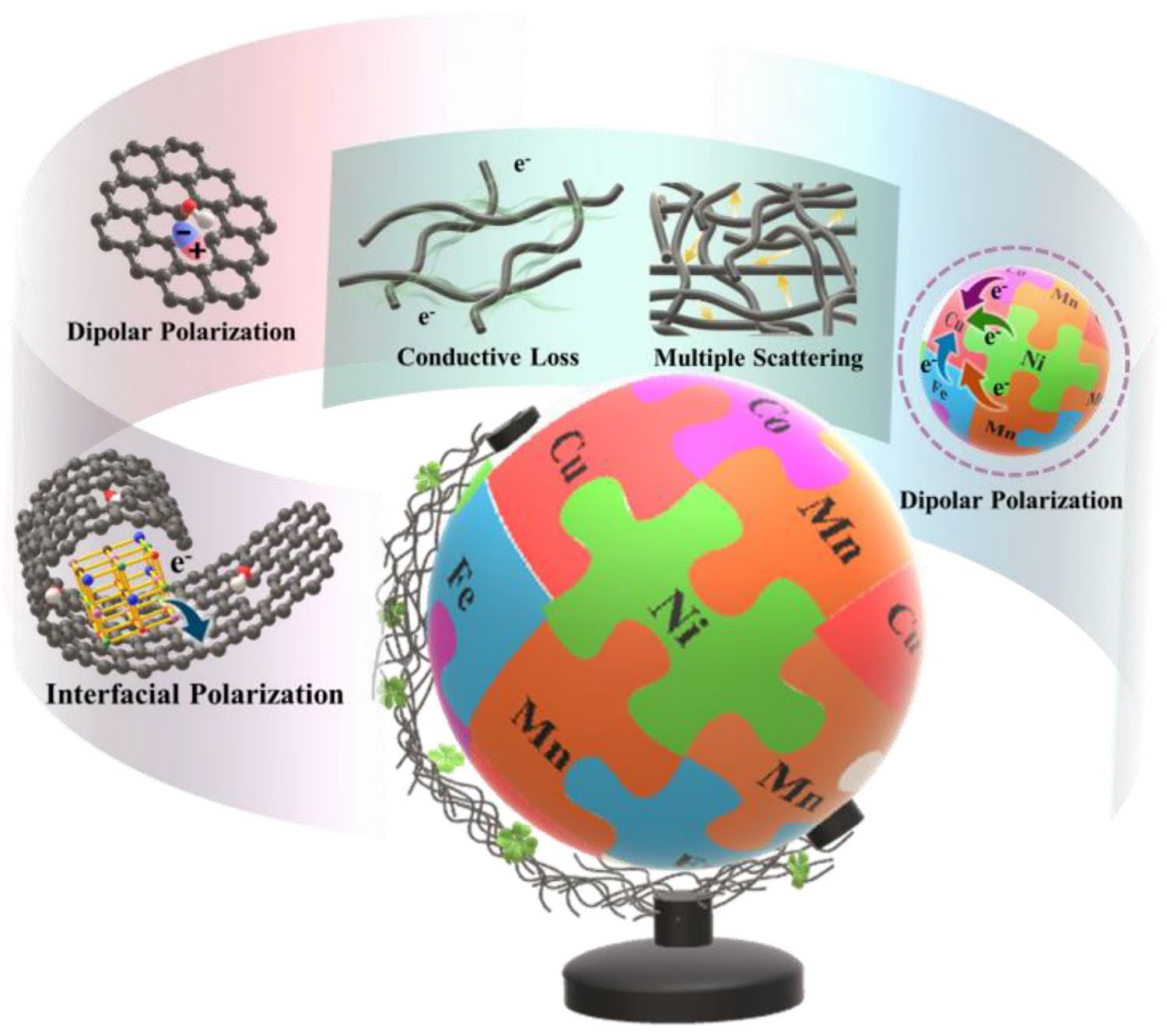
EMW absorbing mechanisms of the CCP/HEAs-Mn_2.15_

The rejuvenated EM response of a system based on biomass-derived carbon/HEAs is implemented by reformative carbothermal shock method and optimized electron migration mode. Firstly, based on the skin-like effect, the integrated carbothermal shock device provides a current protective layer for O· active sites carried by biomass carbon supporter, which can be converted into dipoles to promote dipolar polarization rather than being annihilated during annealing. In addition, the retained O· could act as an active center that absorbs metal precursor droplets to undergo “fission–fusion” nucleation. Meanwhile, O· participates in carbon metabolism as powerfully catalyzed by metals and is released as CO gas that can provide an exogenous driving force for the encapsulation of HEAs with curled CCF, resulting in the formation of emblematic shell-core heterointerface with interfacial polarization. Secondly, randomly distributed metal atoms within HEAs-Mn2.15 have an equal probability of being adjacent to the single electron-rich Cu site, and the accompanying electron migration behavior induces the formation of numerous relatively persistent and localized equivalent dipoles. Thirdly, one-dimensional (1D) CCF with controlled carbonization degree provides fundamental conductive loss for the EMW absorption system to balance impedance matching and attenuation constants [43]. The CCP with 3D conductive network, which is constructed by interleaving CCFs, provides multiple scattering and reflecting spaces for incident EMW. The above-mentioned EMW absorption mechanisms could provide guidelines for the development of multifunctional devices with superior EM response (Fig. 5).

### Multifunctional EM Simulations Based on Real Scenarios

**Fig. 6:**
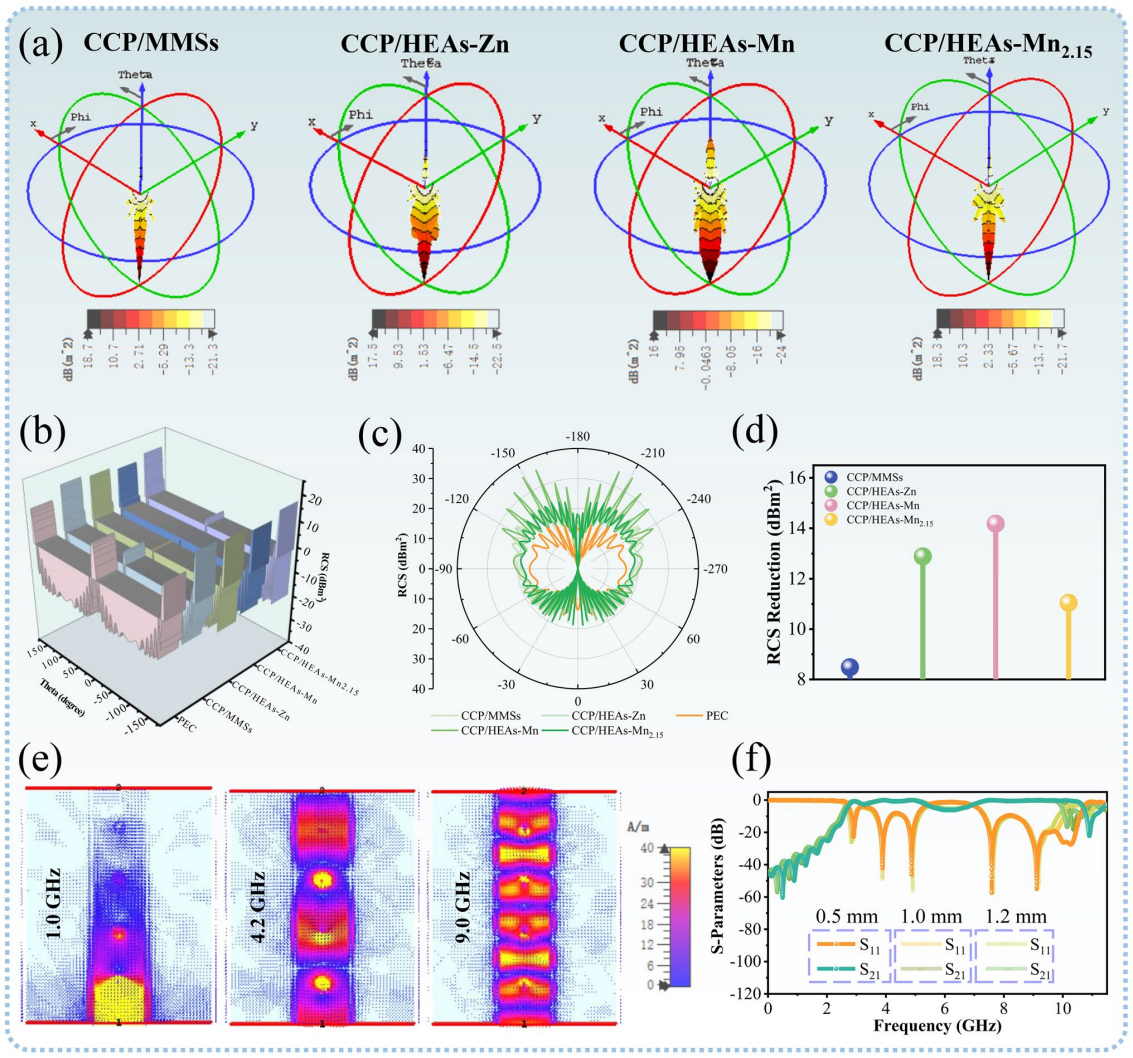
**a** 3D RCS plots, **b** RSC values from − 180° to 180°, **c** RCS in polar coordinate system and **d** RCS reduction values of CCP/MMSs, CCP/HEAs-Zn, CCP/HEAs-Mn and CCP/HEAs-Mn_2.15_; **e** Surface current distributions in passband and stopband and **f** |S11| and |S21| curves of CCP/MMSs-20%

Radar cross section (RCS) is an important parameter for radar detection and identification, stealth and anti-stealth research, which is considered to be the main content of modern military countermeasure technology. RCS simulation is used to predict and evaluate the radar stealth effect under actual far-filed condition. 3D signal intensity mappings are shown in Fig. 6a by collecting radar plane wave scattering signals on a perfect conductor (PEC) coated with the target samples. All samples have different RCS reduction values at corresponding matching thickness, and effective EM attenuation is achieved according to the localized electron migration mechanism of CCP/HEAs-Mn_2.15_ with ultra-thin thicknesses (Fig. 6d). Furthermore, the shape design and the detection angle have an interfering effect on the signal transmission of the radar detector. The RCS reduction values of all samples are maximized at 0° (Figs. 6b, c and S15). The design of strongly coupled EM attenuation units with the aid of localized electron migration effects is a necessary prerequisite for bridging the gap between basic theory and practical radar stealth.

A bandpass filter is an EM component used for the storage, processing and transmission of EMW information. An ideal bandpass filter should have a completely flat passband, where there is no amplification or attenuation of signals within the passband, and all signals outside the passband are completely attenuated. In order to achieve the resonant frequency in the ultra-wide passband, the ultra-wideband (UWB) bandpass filter using CCP/MMSs (with a doping content of 20%, CCP/MMSs-20%) covered with copper transmission line topology as the dielectric substrate is designed and its filtering performances with different thicknesses of substrates are simulated (Fig. S16). When the thickness of dielectric substrate is 0.5 mm, the ultra-wide passband of the filter can reach 7.68 GHz (2.92–10.6 GHz), and the suppression outside the band can reach − 60.5 dB (Fig. 6f). Low insertion loss is attributed to low dielectric loss of CCP/MMSs-20%. Surface current distributions are used to simulate the dynamic transmission of signals at different frequencies. As shown in Fig. 6e, the current in stopband (1.0 GHz) is concentrated at the input port, indicating that the current transmission is suppressed, while the current in passband (4.2 and 9.0 GHz) is transmitted along the topology line from the input port to the output port with undiminished current density, indicating the formation of an effective passband. The design of resonant frequency suitable for UWB filter is achieved by regulating the doping amount and thickness of CCP/MMSs, which provides a promising way for the application of UWB filters in wireless communication, electronic countermeasures, audio and image acquisition.

## Conclusion

In summary, carbothermal shock devices equipped with highly conductive apparatus further extend the choice of carbon source to biomass carbon, which facilitates the retention of active site O· to absorb metal with strong catalytic activity for carbon metabolism, HEAs nucleation and dipole polarization. CCP/HEAs-Mn_2.15_ with the emblematic shell-core heterointerfaces prepared by the reformative carbothermal shock method exhibits promising EMW absorption performance (− 51.35 dB) at an ultra-thin thickness (1.03 mm). The electron migration mode with single electron-rich sites is proved by theoretical calculations to be an effective strategy for the generation of anisotropic equivalent dipoles and the enhancement of the fettering electron ability of HEAs-Mn_2.15_. This work provides a relevant theoretical basis for optimizing dielectric properties of HEAs, which is dominated by electronegativity, valence electron configurations and molar proportions of the constituent elements. Simultaneously, the EM simulation of UWB bandpass filter with CCP/MMSs-20% as dielectric substrate opens up new horizons for the development of multifunctional EM response devices.

## Supplementary Information

Below is the link to the electronic supplementary material.

Supplementary file1 (DOCX 2478 KB)
