# Supplementary material for: Spontaneous Orientation Polarization of Anisotropic Equivalent Dipoles Harnessed by Entropy Engineering for Ultra-Thin Electromagnetic Wave Absorber
Source: Nanomicro Lett. 2024 Sep 26;17:19. doi: 10.1007/s40820-024-01507-0 (PMC11427666; doi:10.1007/s40820-024-01507-0)
Supplement: Supplementary file 1 — Supplementary file1 (DOCX 2478 KB) [file 40820_2024_1507_MOESM1_ESM.docx]

Supporting Information for

**Spontaneous Orientation Polarization of Anisotropic Equivalent Dipoles Harnessed by Entropy Engineering for Ultra-Thin Electromagnetic Wave Absorber**

Honghan Wang^1,^†, Xinyu Xiao^1,^†, Shangru Zhai^1,^*, Chuang Xue^2,^*, Guangping Zheng^3^, Deqing Zhang^4^, Renchao Che^5,^*, Junye Cheng^6,^*

^1^Liaoning Key Lab of Lignocellulose Chemistry and BioMaterials, Liaoning Collaborative Innovation Center for Lignocellulosic Biorefinery, School of Light Industry and Chemical Engineering, Dalian Polytechnic University, Dalian 116034, P. R. China

^2^School of Life Science and Biotechnology, Dalian University of Technology, Dalian 116024, P. R. China

^3^Department of Mechanical Engineering, Hong Kong Polytechnic University, Hung Hom, Kowloon, Hong Kong 999077, P. R. China

^4^School of Materials Science and Engineering, Qiqihar University, Qiqihar 161006, P. R. China

^5^Laboratory of Advanced Materials, Shanghai Key Lab of Molecular Catalysis and Innovative Materials, Academy for Engineering & Technology, Fudan University, Shanghai 200438, P. R. China

^6^Department of Materials Science, Shenzhen MSU-BIT University, Shenzhen 517182, P. R. China

†Honghan Wang and Xinyu Xiao contributed equally to this work.

*Corresponding authors. E-mail: [zhaisrchem@163.com](mailto:zhaisrchem@163.com) (Shangru Zhai); [xue.1@dlut.edu.cn](mailto:xue.1@dlut.edu.cn) (Chuang Xue); [rcche@fudan.edu.cn](mailto:rcche@fudan.edu.cn) (Renchao Che); [chengjunye@smbu.edu.cn](mailto:chengjunye@smbu.edu.cn) (Junye Cheng)

S1 Experimental Details

S1.1 Synthesis of CCP

80 g poplar wood chips were placed inside a steam tank filled with 1000 mL mixture of ethanol and water (the ratio of ethanol to water is 4:1), which was steamed at 180 °C for 4 h. The separated celluloses were made into 0.1 mm paper scraps using a paper machine. Subsequently, paper scraps were pre-carbonized in a tube furnace (200 °C for 6 h for the first time and 800 °C for 3 h for the second time). The obtained samples were labeled as CCP.

S1.2 Characterizations

The X-ray diffraction patterns was recorded using Shimadzu XRD-7000S X-ray diffractometer with Cu Kα radiation (λ=1.5406 Å). Raman spectra were recorded by Raman spectroscopy (UK, Renishaw-InVia Basis). XPS analysis was implemented on X-ray photoelectron spectrometer (Thermo Scientific K-Alpha). The surface morphology was observed by scanning electron microscopy (SEM, JSM-7800F) equipped with an EDS apparatus (UK, Oxford, X-Max50). The microstructure was characterized by transmission electron microscopy (TEM) and a high-resolution transmission electron microscope (HRTEM) (JEM-2100(UHR)). The electrical conductivity was measured by an ST2253y four-probe resistivity meter. The EM parameters were collected using a vector network analyzer (VNA, Agilent E5071C) via the coaxial method in the 2~18 GHz range. The mixture of the sample and paraffin wax was pressed into a coaxial ring with a thickness of 2 mm using a mold, and the doping content of the samples was 55%, except for 20% of CCP/MMSs.

S1.3 EM Parameters and EMW Absorption Performance Calculation

To analyze the dependence of EM response mode on frequency in the 2-18 GHz range, EM parameters are measured by the coaxial method using an Agilent E5071C vector network analyzer. The dielectric loss and the magnetic loss determined by the EM parameters as characterized by the relative complex permittivity (ε_r_) and relative complex permeability (µ_r_), respectively, mainly dominate EMW attenuation. The ε_r_ can be expressed as follows:

$\varepsilon_{r}=\varepsilon_{\infty}+\frac{\varepsilon_{s}-\varepsilon_{\infty}}{1+j2\pi f\tau}=\varepsilon^{'}-j\varepsilon^{''}$ . (S1)

The terms of ε' is the real part of permittivity associated with energy storage. On the contrary, ε'' is the imaginary part of permittivity related to the energy dissipation. Then ε' and ε" could be described as follows:

$\varepsilon^{'}=\varepsilon_{\infty}+\frac{\varepsilon_{s}-\varepsilon_{\infty}}{1+\omega^{2}\tau^{2}}=\varepsilon_{\infty}+\frac{\varepsilon_{s}-\varepsilon_{\infty}}{1+{(2\pi f)}^{2}\tau^{2}}$ , (S2)

$\varepsilon^{''}=\frac{\varepsilon_{s}-\varepsilon_{\infty}}{1+\omega^{2}\tau^{2}}\omega\tau+\frac{\sigma}{\omega\varepsilon_{0}}=\frac{2\pi f\tau(\varepsilon_{s}-\varepsilon_{\infty})}{1+{(2\pi f)}^{2}\tau^{2}}$ , (S3)

where ω is the angular frequency, τ refers to the polarization relaxation time, ε_0_ is the dielectric constant in vacuum (8.854×10^-12^ F·m^-1^), ε_s_ means the static permittivity, ε_∞_ is the permittivity at an infinite frequency, and σ is conductivity.

The degree of dielectric loss affected by dielectric properties is determined by the tangent of the dielectric loss angle (tanδε=ε″/ε′). The dielectric frequency dispersion is strongly associated with polarization loss and conductive loss. According to the free electron theory, σ can directly affect ε″:

$\varepsilon^{''}\approx\sigma/2\pi\varepsilon_{0}f$ . (S4)

Thus, a larger σ value leads to a larger ε" value, which increases the conductive loss. The dipole polarization can be explained by the Cole-Cole formula as follows:

${(\varepsilon^{'}-\frac{\varepsilon_{s}+\varepsilon_{\infty}}{2})}^{2}+{(\varepsilon")}^{2}={(\frac{\varepsilon_{s}-\varepsilon_{\infty}}{2})}^{2}$ . (S5)

According to the Debye theory, εʺ can be clarified as follows:

$\varepsilon"\text{=}\text{ε}_{\text{p}}\text{"+}\text{ε}_{\text{c}}"=\frac{\varepsilon_{s}-\varepsilon_{\infty}}{1+\omega^{2}\tau^{2}}\omega\tau+\frac{\sigma}{\omega\varepsilon_{0}}$ . (S6)

According to Eq. (S3), the polarization relaxation time can be expressed as follows:

$\varepsilon^{'}=\frac{1}{2\pi\tau}\frac{\varepsilon"}{f}+\varepsilon_{\infty}$ . (S7)

The relationship between ε′ and ε″/f is a straight line with a slope of 1/2πτ, from which the relaxation time τ for polarization relaxation can be calculated.

According to transmission line theory, the value of reflection loss (RL) characteristics of EMW absorbing materials, which directly reflect their EM absorption performance, can be expressed as follows:

$Z_{\mathrm{in}}=Z_{0}\sqrt{\frac{\mu_{r}}{\varepsilon_{r}}}\tanh\left[ \frac{2\pi jfd}{c}\sqrt{\mu_{r}\varepsilon_{r}} \right]$ . (S8)

On the basis of Eq. S9, Eq. S10 is the numerical solution obtained by Steffensen's acceleration method:

$RL=20lg\left| \frac{(Z_{\mathrm{in}}-Z_{0})}{(Z_{\mathrm{in}}+Z_{0})} \right|$ , (S9)

where f is the frequency, d is the matching thickness, c is the velocity of light in vacuum (3×10^8^ m·s^-1^), and Z_0_ and Z_in_ are the free space impedance and the normalized input impedance of absorbers, respectively.

The impact of impedance matching (M_Z_) and attenuation constants (α) on the EM attenuation capacity is important. M_Z_ represents the ability of incident EMW to enter into the absorber from free space, while the value of α is used to evaluate the ability of EMW to be attenuated after entering the absorber. An excellent impedance matching condition can be expressed as follows:

$\left| Z_{\mathrm{in}}/Z_{0} \right|=\left| \sqrt{\frac{\mu_{r}}{\varepsilon_{r}}}\tanh\left[ \frac{2\pi jfd}{c}\sqrt{\mu_{r}\varepsilon_{r}} \right] \right|$ . (S10)

The attenuation constant is usually expressed as follows:

$\alpha=\frac{\sqrt{2}\pi f}{c}\sqrt{\left( \mu_{r}^{''}\varepsilon_{r}^{''}-\mu_{r}^{'}\varepsilon_{r}^{'} \right)+\sqrt{\left( \mu_{r}^{''}\varepsilon_{r}^{''}-\mu_{r}^{'}\varepsilon_{r}^{'} \right)^{2}+\left( \mu_{r}^{'}\varepsilon_{r}^{''}+\mu_{r}^{''}\varepsilon_{r}^{'} \right)^{2}}}$ . (S11)

Typically, if M_z_ at a peak in the M_z_ versus frequency plots is closer to 1, the absorber has a more suitable impedance matching. Meanwhile, a larger value of α represents better EM attenuation capability.

S1.4 Fundamental Parameters Related to HEAs Formation

Based on the rules and thermodynamic consideration, the formation of HEAs is mainly influenced by mixing entropy, mixing enthalpy and atomic size diﬀerence.

The mixing entropy (ΔS_mix_) of HEAs can be approximated as the configurational entropy (ΔS_conf_), which can be formulated as follows:

$\Delta S_{mix}=\Delta S_{conf}=-R\sum_{i=1}^{n} c_{i}lnc_{i}$ (S12)

where R represents the mole gas constant, and c_i_ is the atom percentage of the i^th^ component, and n is the number of components in the alloys.

The mixing enthalpy of HEAs can be expressed as follows:

$\Delta H_{mix}=\sum_{i=1, i\neq j}^{n} 4\Delta H_{ij}^{mix}c_{i}j_{i}$ (S13)

where c_i_ or c_j_ is the atomic percentage of the i^th^ or j^th^. Δ$H_{ij}^{mix}$ represents the mixing enthalpy of the atom pair between the i^th^ and j^th^ components.

In addition to thermodynamic parameters, atomic size diﬀerence (δ), as a topological parameter, is also closely related to the stability of HEAs, whose calculated formula is shown as follows:

$\delta=\sqrt{\sum_{i=1}^{n} c_{i}\left( 1-r_{i}/\sum_{i=1}^{n} c_{i}r_{i} \right)^{2}}$ (S14)

Where r_i_ and c_i_ are the atomic radii and atomic percentage of the i^th^ element, respectively.

S1.5 RCS Simulation

Frequency domain solver is used in the RCS simulation process. The simulation models are constructed by a top absorber and a bottom perfect conductive layer PEC. The dielectric constant and permeability of the absorbers are used as the EM parameters for input material, and the matched thickness is used as the thickness parameter of the material. The monitor setting frequency is matched to the RL_min_ frequency. Specifically, the simulations based on the far-field conditions of 17.68, 15.36, 12.72 and 16.96 GHz corresponding to RL_min_ of L-CFP/FeCoNiCuZn-X are implemented for PEC substrates (180 × 180 × 1 mm) coated with the above-mentioned three samples with 4.01, 4.00, 5.04, 1.03 mm, respectively, when EMW is incident along the z-axis (detection angle of 0°). Scattered signal intensity is used as a measure of EMW absorption capacity.

S1.6 Ultra-Wideband Bandpass Filter Simulation

The ultra-wideband bandpass filter simulation results are obtained using CST 3D electromagnetic field simulation software.

S1.7 DFT Calculation

The structural optimization is carried out by spin-polarized density functional theory (DFT) calculations using generalized-gradient-approximation (GGA) with the Perdew Burke Ernzerhof (PBE) exchange-correlation functional. Double numerical plus d-functions (DND) is selected as the base to ensure that the accuracy is within an acceptable range. The 2D Brillouin zone is sampled via setting a 3×3×3 Monkhorst-Pack special k-point grid. The convergence tolerances for geometry optimizations are set, which include a maximum force of 0.004 Ha/Å, a maximum displacement of 0.005 Å and energy restriction of 2×10^-5^ Ha.

S2 Supplementary Figures and Tables


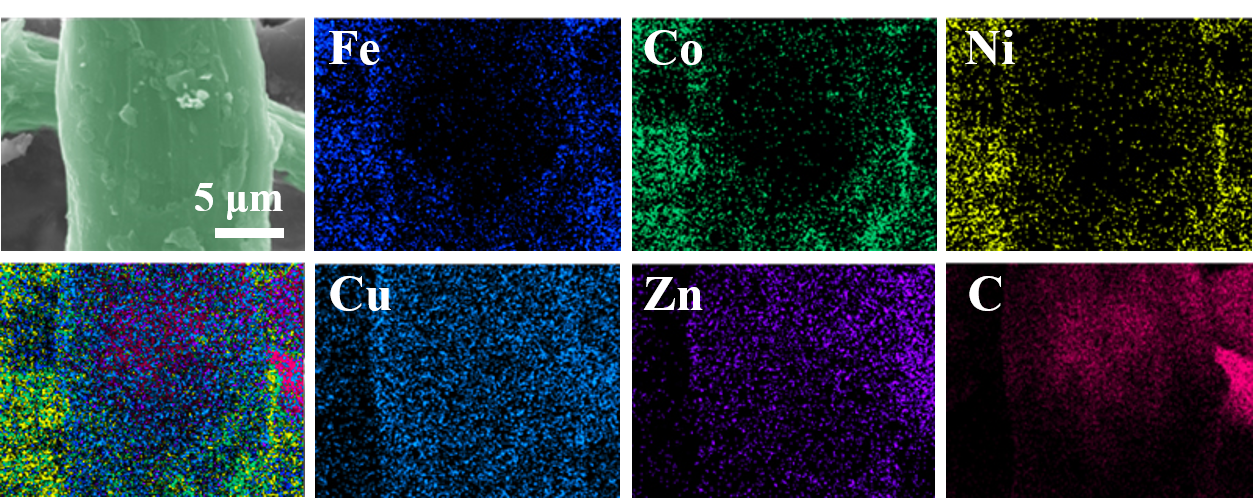


**Fig. S1** Elemental mapping images of CCP/MMSs


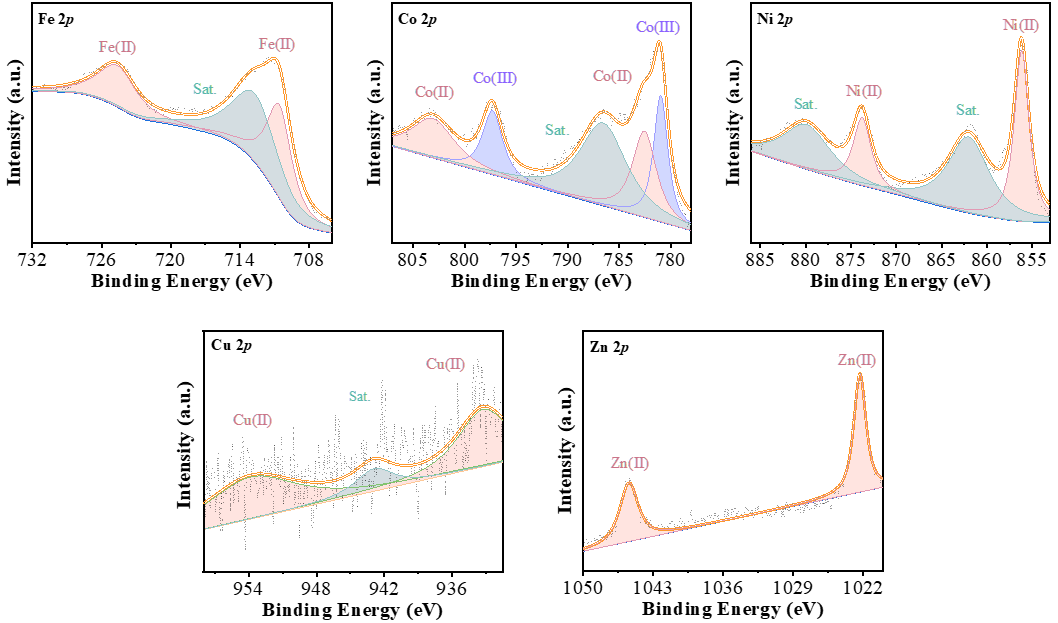


**Fig. S2** XPS spectra of CCP/MMSs


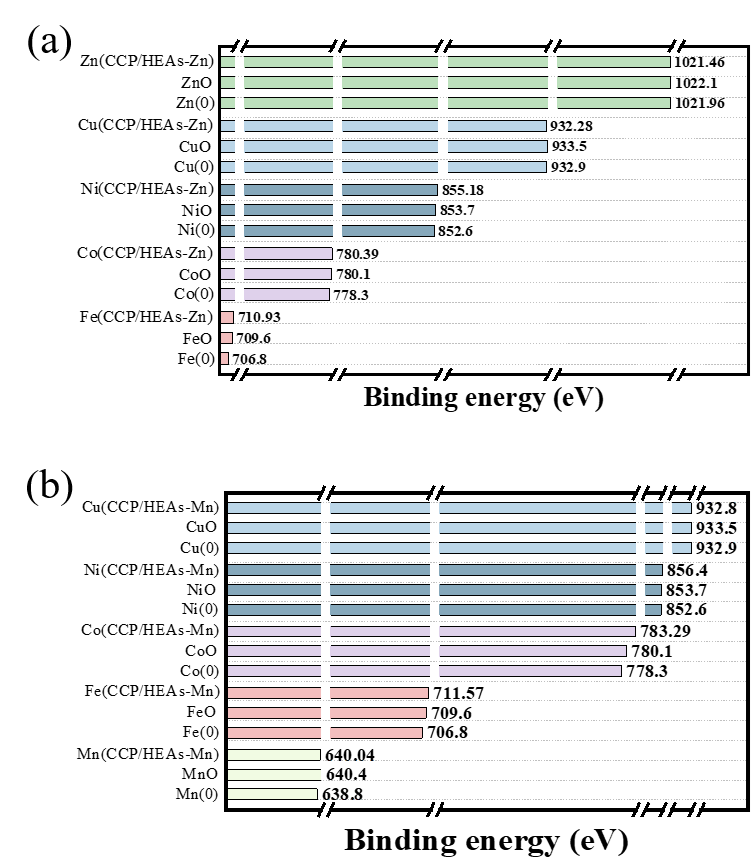


**Fig. S3** A comparison of the binding energies of the HEAs nanoparticles, metals and metal oxides for each element in **(a)** CCP/HEAs-Zn and **(b)** CCP/HEAs-M


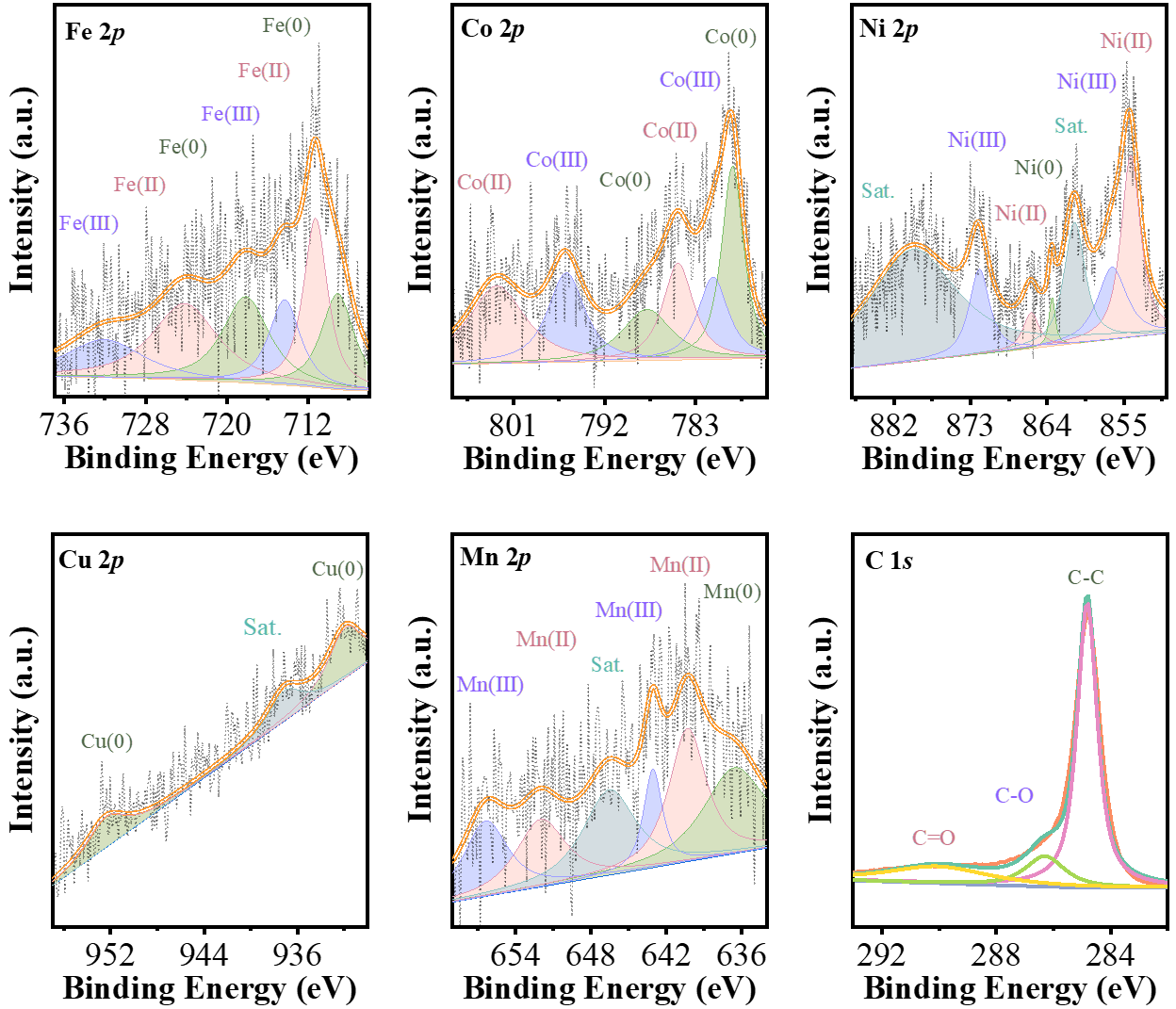


**Fig. S4** XPS spectra of CCP/HEAs-Mn_2.15_


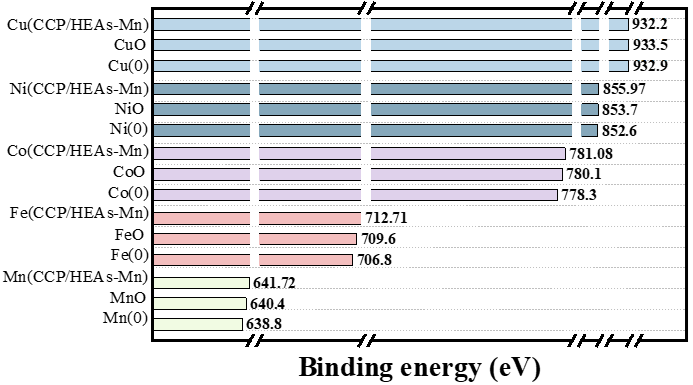


**Fig. S5** A comparison of the binding energies of the HEAs nanoparticles, metals and metal oxides for each element in CCP/HEAs-Mn_2.15_


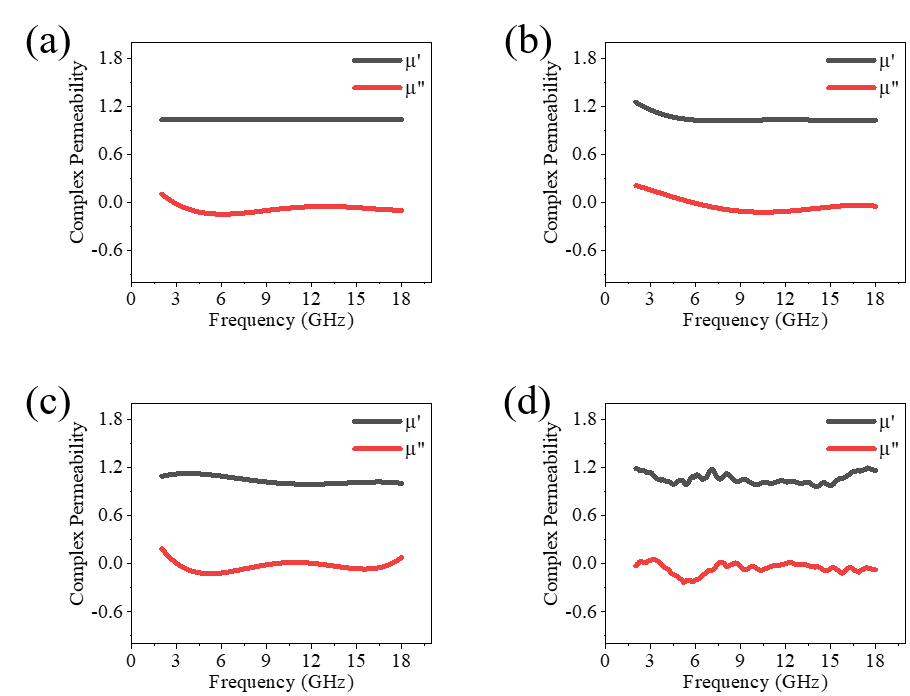


**Fig. S6** The complex permeability of (**a**) CCP/MMSs, (**b**) CCP/HEAs-Zn, (**c**) CCP/HEAs-Mn and (**d**) CCP/HEAs-Mn_2_


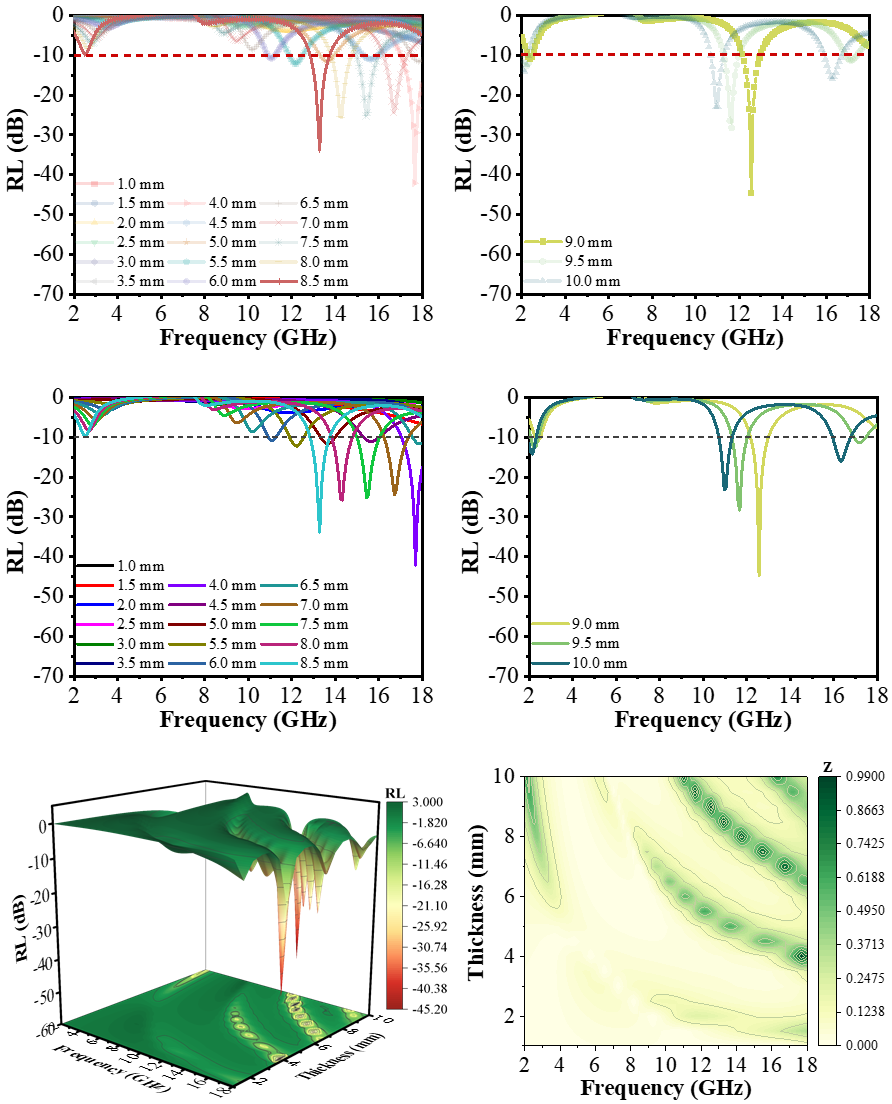


**Fig. S7** 2D/3D RL plots and M_z_ of CCP/MMSs


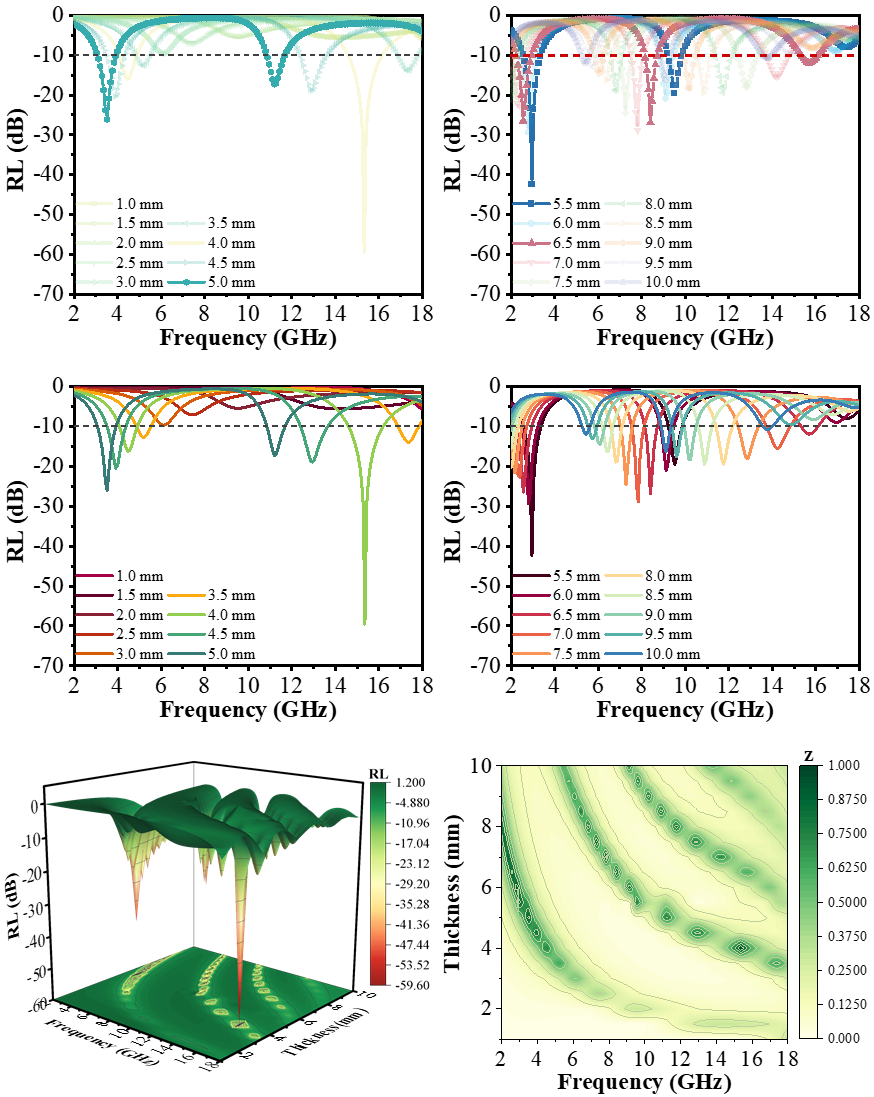


**Fig. S8** 2D/3D RL plots and M_z_ of CCP/HEAs-Zn


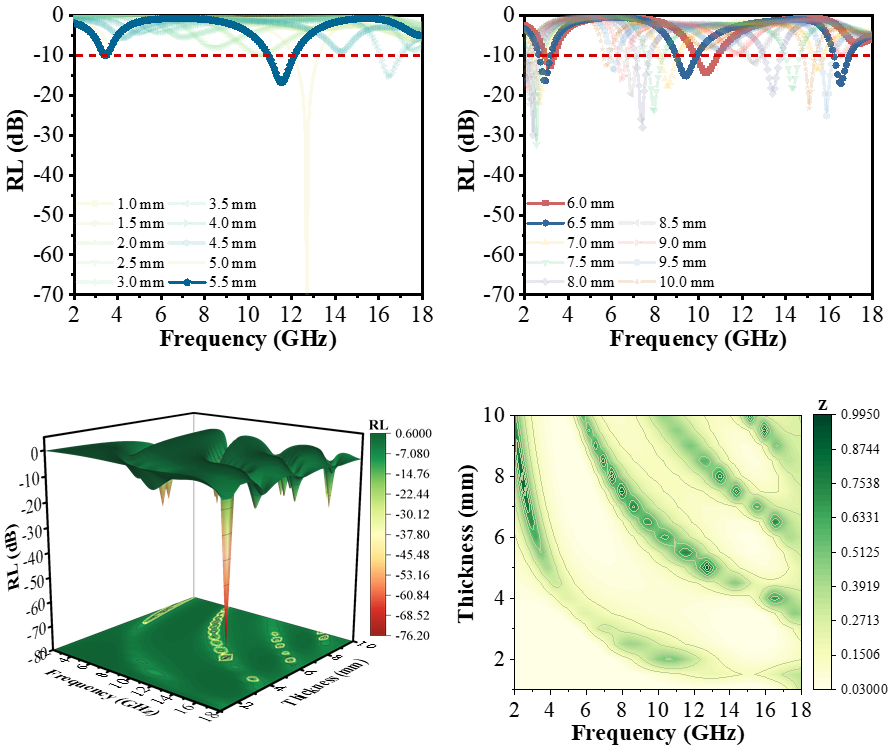


**Fig. S9** 2D/3D RL plots and Mz of CCP/HEAs-Mn


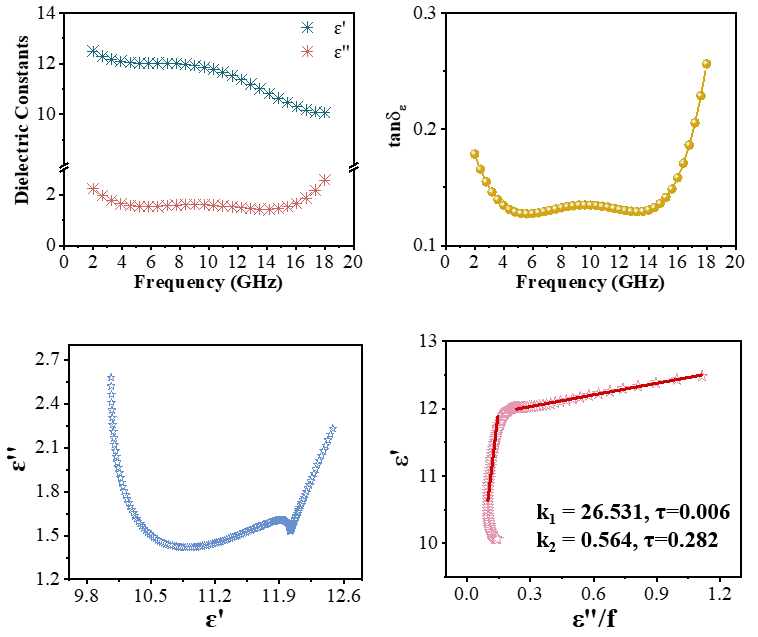


**Fig. S10** The dielectric constants, tanδε, Cole-Cole plots and relaxation time of CCP/MMSs


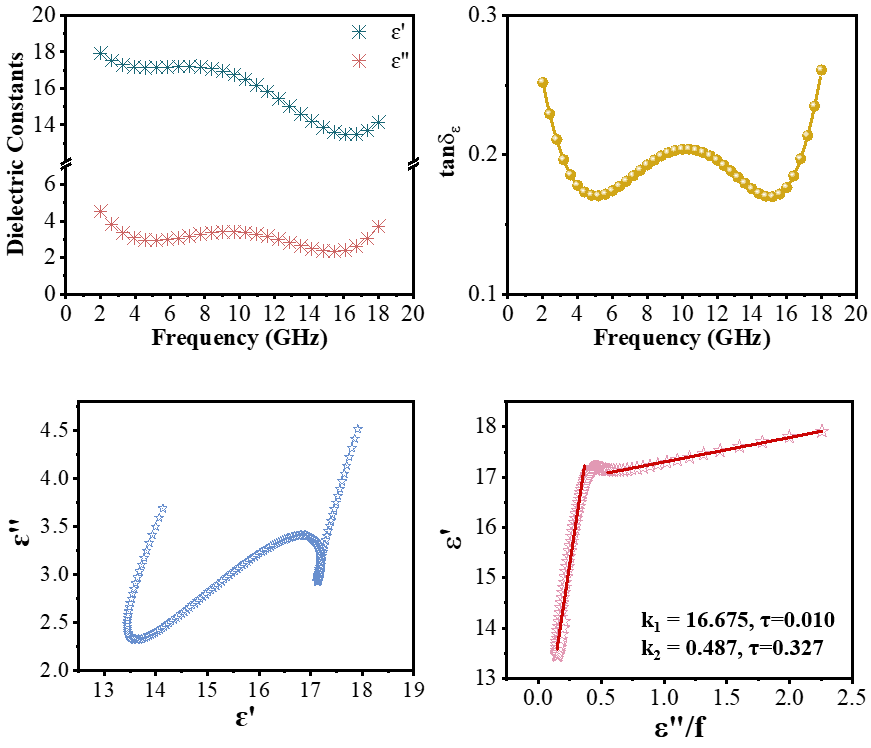


**Fig. S11** The dielectric constants, tanδε, Cole-Cole plots and relaxation time of CCP/HEAs-Zn


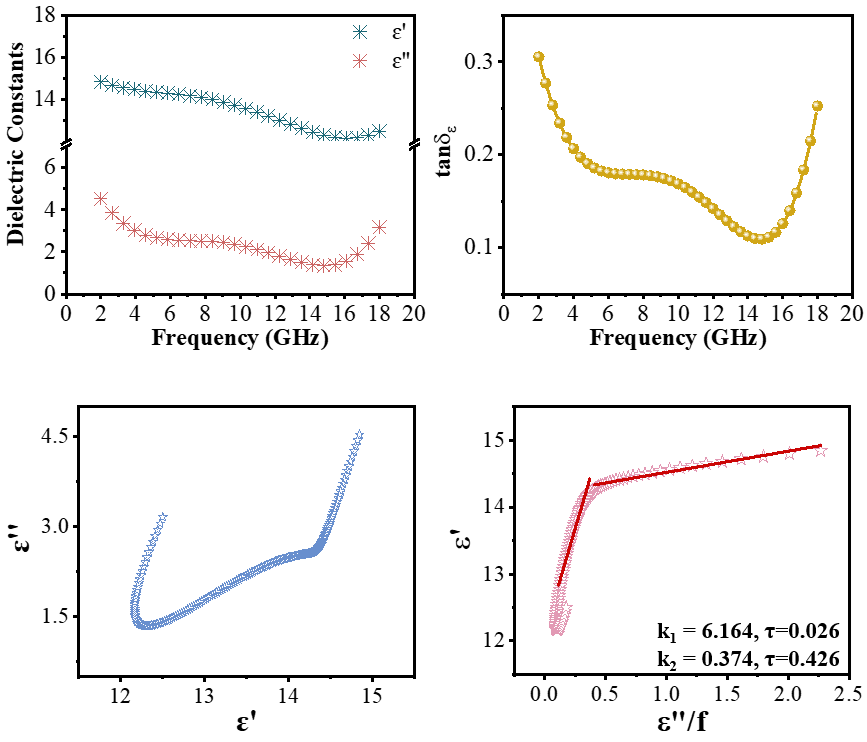


**Fig. S12** The dielectric constants, tanδε, Cole-Cole plots and relaxation time of CCP/HEAs-Mn


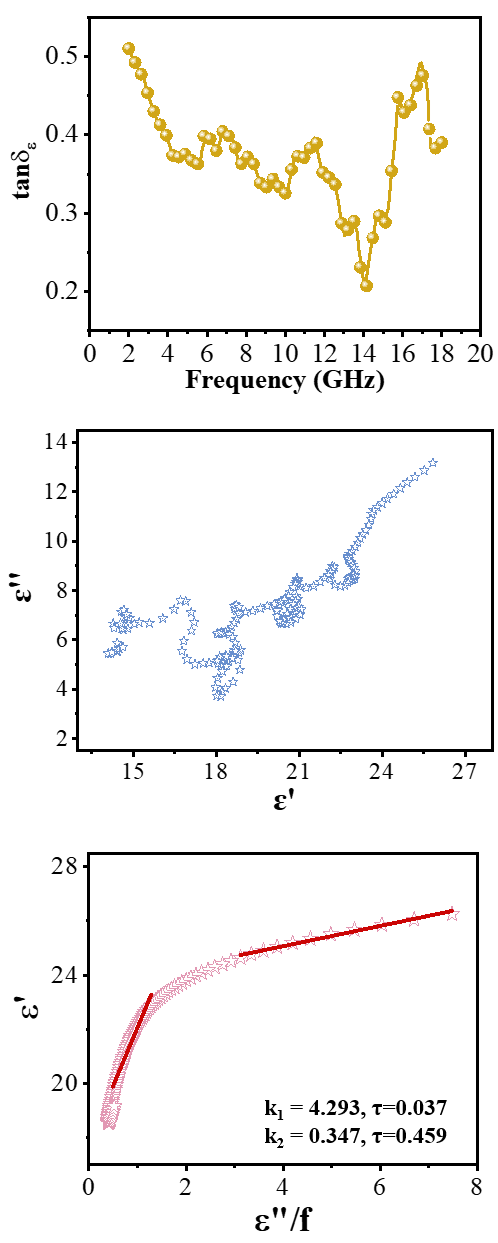


**Fig. S13** tanδε, Cole-Cole plots and relaxation time of CCP/HEAs-Mn_2.15_


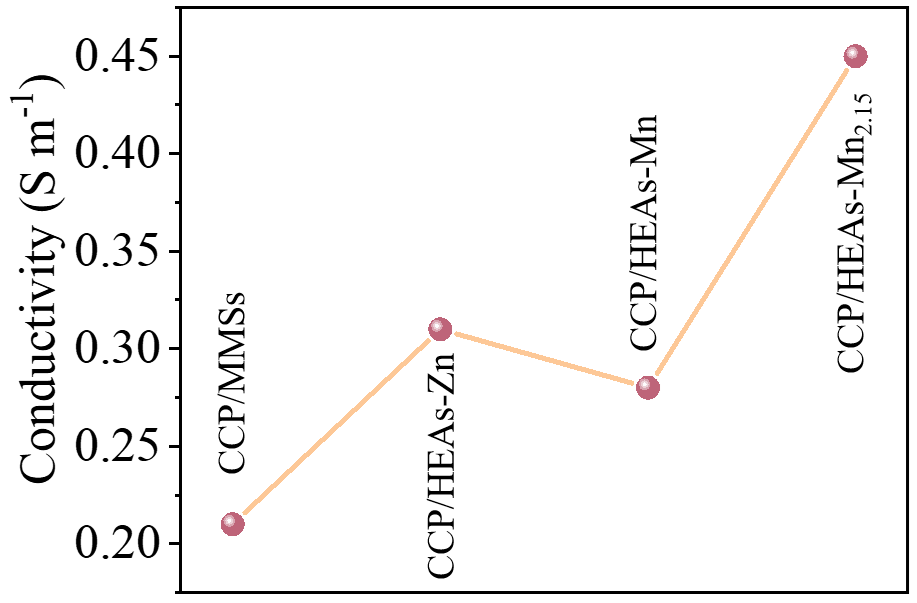


**Fig. S14** The conductivity of CCP/MMSs, CCP/HEAs-Zn, CCP/HEAs-Mn and CCP/HEAs-Mn_2.15_

**Fig. S15** 2D plots of RSC values from -150° to 150°


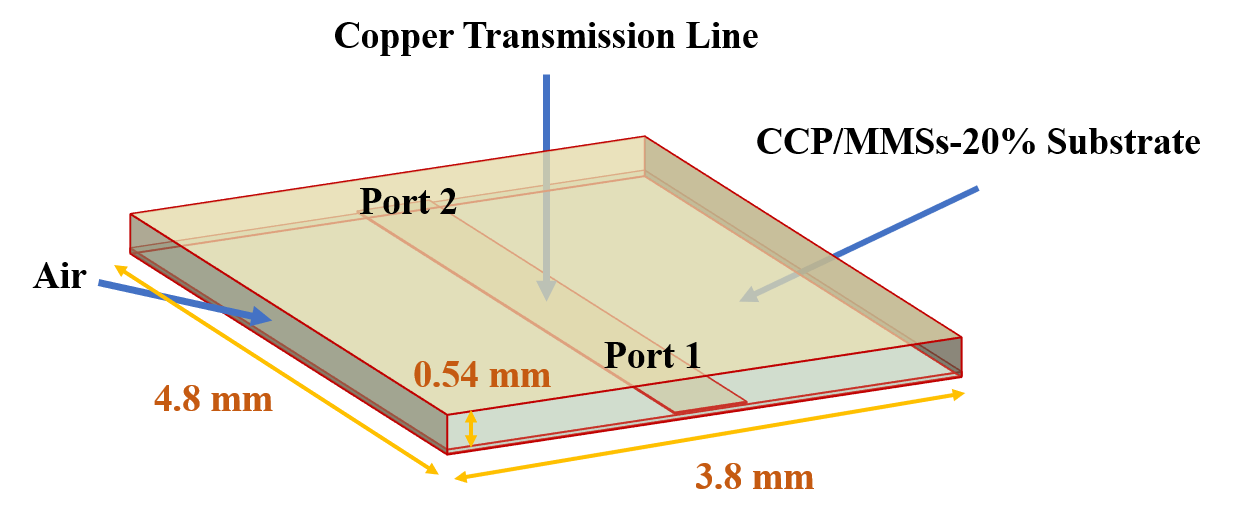


**Fig. S16** The UWB ﬁlter model

**Fig. S17** 2D RL plots of CCP/HEAs-Mn_2.15_ under the shock duration of 1000 ms. The CCP/HEAs_2.15_ under the extended shock duration does not exhibit the ultra-thin electromagnetic wave absorption properties

**Fig. S18** 2D RL plots of CCP/FeCoNi-alloy exhibits thicker matching thickness and lower absorption intensity

**Table S1** Physicochemical properties of Mn, Fe, Co, Ni, Cu, Zn

|  | Mn | Fe | Co | Ni | Cu | Zn |
| --- | --- | --- | --- | --- | --- | --- |
| Atomic Radius (pm) | 117 | 117 | 116 | 115 | 117 | 125 |
| Electronegativity | 1.55 | 1.80 | 1.88 | 1.91 | 1.90 | 1.65 |
| Valence Electron Arrangement | 3d^5^4s^2^ | 3d^6^4s^2^ | 3d^7^4s^2^ | 3d^8^4s^2^ | 3d^10^4s^1^ | 3d^10^4s^2^ |
| Melting Point (K) | 1517.15 | 1811.15 | 1773.15 | 1728.15 | 1356.55 | 692.15 |

**Table S2** The configurational entropy of HEAs-Zn, HEAs-Mn and HEAs-Mn_2.15_

|  | HEAs-Zn | HEAs-Mn | HEAs-Mn_2.15_ |
| --- | --- | --- | --- |
| ΔS_mix_ | 1.61R | 1.61R | 1.55R |

**Table S3** Performance comparison with published cutting-edge works in terms of matching thickness

| Refs. | Materials | Matching Thickness |
| --- | --- | --- |
| [S1] | PCS-F | 1.60 mm |
| [S2] | FeCoNiCuC_0.37_ | 1.95 mm |
| [S3] | MCFC-69-8 | 1.48 mm |
| [S4] | Fe_x_N@NGC/Ce | 1.50 mm |
| [S5] | CoNiM@C | 2.00 mm |
| This Work | CCP/HEAs-Mn_2.15_ | 1.03 mm |

**Supplementary References**

1. A. Elhassan, J. Li, I. Abdalla, Z. Xu, J. Yu, Z. Li, B. Ding, Ant-nest-inspired biomimetic composite for self-cleaning, heat-insulating, and highly efficient electromagnetic wave absorption. Adv. Funct. Mater. 2407458 (2024). <https://doi.org/10.1002/adfm.202407458>
2. Z. Qiu, X. Liu, T. Yang, J. Wang, Y. Wang, W. Ma, Y. Huang, Synergistic enhancement of electromagnetic wave absorption and corrosion resistance properties of high entropy alloy through lattice distortion engineering. Adv. Funct. Mater. 2400220 (2024). <https://doi.org/10.1002/adfm.202400220>
3. J. Wang, L. Zhang, J. Yan, J. Yun, W. Zhao, K. Dai, H. Wang, Y. Sun, MXene-based ultrathin electromagnetic wave absorber with hydrophobicity, anticorrosion, and quantitively classified electrical losses by intercalation growth nucleation engineering. Adv. Funct. Mater. 2402419 (2024). <https://doi.org/10.1002/adfm.202402419>
4. Z. Ma, K. Yang, D. Li, H. Liu, S. Hui, Y. Jiang, S. Li, Y. Li, W. Yang, H. Wu, Y. Hou, The electron migration polarization boosting electromagnetic wave absorption based on Ce atoms modulated yolk@shell FexN@NGC. Adv. Mater. **36**, 2314233 (2024). <https://doi.org/10.1002/adma.202314233>
5. M. Huang, B. Li, Y. Qian, L. Wang, H. Zhang, C. Yang, L. Rao, G. Zhou, C. Liang, R. Che, MOFs‑derived strategy and ternary alloys regulation in flower‑like magnetic‑carbon microspheres with broadband electromagnetic wave absorption. Nano-Micro Lett. **16**, 245 (2024). <https://doi.org/10.1007/s40820-024-01416-2>
